# An empirically-based scenario for the evolution of cultural transmission in the human lineage during the last 3.3 million years

**DOI:** 10.1371/journal.pone.0325059

**Published:** 2025-06-04

**Authors:** Ivan Colagè, Francesco d’Errico

**Affiliations:** 1 Faculty of Philosophy and DISF Research Centre, Pontifical University of the Holy Cross, Rome, Italy; 2 Université de Bordeaux, Department of Archeological Science, CNRS, Unité Mixte de Recherche 5199 PACEA, Pessac, France; 3 Department of Archaeology, History, Cultural Studies and Religion, Centre for Early Sapiens Behaviour (SapienCE), University of Bergen, Bergen, Norway; Sapienza University of Rome: Universita degli Studi di Roma La Sapienza, ITALY

## Abstract

Humans accumulate an ever-growing body of knowledge that far exceeds the capacity of any single individual or generation. Social learning and transmission are essential for this process. However, how cultural transmission strategies evolved in our lineage remains unclear. Here we assess the transmission strategies needed to ensure the perpetuation across generations of 103 cultural traits that emerged in the Paleolithic. Our study provides a novel approach to assessing the transmission behaviors implicated in Paleolithic cultural traits and the evolution of cultural transmission over the last 3.3 million years. The results identify trends in the evolution of cultural transmission and reveal a coevolutionary dynamic between the emergence of novel cultural traits and the complexification of transmission strategies. While effective means of overt explanation, perhaps associating gesture and verbal expression, were already present at least 600,000 years ago, the period between 200,000 and 100,000 years ago appears as a crucial tipping point for the emergence of modern language.

## Introduction

Culture has long been considered a human-specific trait. It has been defined as “information capable of affecting individuals’ behavior that they acquire from other members of their species through teaching, imitation, and other forms of social learning” [1, p. 5], or as “group-typical behavior patterns shared by members of a community that rely on socially learned and transmitted information” [2, p. 151]. Defined this way, culture turns out *not* to be exclusively human, as many animal species display forms of culture and behavioral traditions [3 for review].

*Cumulative* culture is somewhat different. It can be understood as “a process by which innovations are progressively incorporated into a population’s stock of skills and knowledge, *generating more complex repertoires*” [4, p. 7877]. An important aspect of cumulative culture is what has been called the “ratchet effect” [5], according to which a culture is truly cumulative if it is able to accumulate innovations over time by building upon previously devised behavior, strategies, or artefacts [6].

Cumulative cultural evolution (CCE) implies that a culture can “accumulate changes over many generations, resulting in culturally transmitted behaviors that *no single human individual could invent on their own*” [7 p. 80; see also 8,9]. Cultural novelty may be built either by chaining together different elements, recombining existing elements in novel behavior and product, or repurposing existing elements in view of new outcomes [10,11]. This understanding of cumulative cultural evolution is not readily applicable to non-human animal cultures, whereas it may hold for a number of hominin cultures [12]. A peculiarity of human culture is the capacity to implement two different kinds of cumulative cultural evolution [13]: the ability to optimize exploitation of specific natural phenomena (Type I CCE), and the ability to progressively recruit additional natural phenomena (Type II CCE). It is unclear whether both, just one, or neither of these two types of cumulative cultural evolution is peculiar to our species, *Homo sapiens*. Indeed, since our last common ancestor with the genus *Pan*, which lived 5–7 million years ago, paleoanthropologists have identified dozens of genera, species, subspecies, and regional variants within our lineage and clade (the *Hominina* subtribe), along with a myriad of cultural adaptations in different paleo-environments.

The evolution of cultural transmission, a cornerstone of human adaptation, remains a subject of intense debate. Tennie and colleagues [14] argue that cumulative culture uniquely distinguishes humans from other species, proposing the *Zone of Latent Solutions* framework to identify behaviors reliant on social learning. Complementing this perspective, Stout and colleagues [15] demonstrate how the neurological underpinnings of toolmaking display feedback loops between cognition and culture. Van Schaik and colleagues [16] further emphasized the ecological and social prerequisites for cumulative culture, arguing that resource-rich environments and complex social networks were critical drivers of its emergence. These factors are particularly relevant to understanding the transitions observed in hominin cultural adaptations over the past 3.3 million years, from the Oldowan tool tradition to the sophisticated material cultures of the Upper Paleolithic.

More recently, Paige and Perreault [17] have applied computational models to elucidate the evolutionary dynamics of cultural transmission, highlighting how innovations spread through populations and are retained across generations. Their work underscores the importance of quantitative frameworks in tracing the trajectory of cultural evolution over deep time. Shipton [18] challenges prevailing assumptions about the universality of cumulative culture in the Paleolithic, arguing that while certain technological traditions exhibit elements of accumulation, others reflect stasis or cyclical innovation. His analysis highlights the need to consider variability in cultural transmission and innovation within the Paleolithic record, emphasizing that cumulative culture may not have been as widespread or uniform as often assumed. In contrast, Stibbard-Hawkes [19] challenges the assumption that the presence of symbolic material culture in the archaeological record directly reflects the cognitive capacities of past populations. By analyzing the material culture of contemporary African forager groups, he demonstrates that behaviorally modern societies may leave minimal enduring evidence of symbolic behavior due to factors such as residential mobility and material choices. This insight underscores the need for caution when interpreting the archaeological record and suggests that the absence of certain cultural artifacts does not necessarily imply a lack of cognitive complexity in ancient populations. This issue is further complicated by the role of taphonomic bias in shaping the archaeological record. Surovell et al. [20] developed a method for correcting temporal frequency distributions to account for differential preservation, emphasizing that raw artifact counts alone may misrepresent patterns of cultural change. More recently, Kelly, Mackie, and Kandel [21] tested whether the rapid increase in symbolic artifact production after 45,000 years ago was merely a consequence of preservation bias, concluding that this pattern reflects a genuine cultural phenomenon rather than an artifact of differential taphonomic processes. These studies highlight the necessity of integrating quantitative and taphonomic considerations when assessing the timing and intensity of cultural innovations in the deep past.

Building on these insights, the aim of the present paper is to elaborate an empirically based scenario for the evolution of cultural transmission in our lineage that identifies at a fine-grained scale when and how we have moved to the transmission strategies attested in current human cultures. This scenario explores the interplay between the emergence of more and more complex cultural traits and the mechanisms triggering a parallel complexification of modes of cultural transmission.

Virtually every scholar inquiring into cultural evolution from the fields of behavioral ecology, ethology, evolutionary biology, physical anthropology, psychology and neuroscience agrees that culture entails *social learning*, i.e., learning from others – often, conspecifics. Asocial learning (i.e., learning on one’s own without any social cue) is considered to play a role [22,23], especially as a factor introducing novel solutions and innovations necessary to burst the cultural process. True, learning is rarely purely social or purely individual but rather a hybrid process, combining trial and error with social input in varying degrees. While trial-and-error learning plays a key role in cultural transmission, we argue that it is most effective when embedded within a structured cultural scaffolding—that is, conditions that guide, facilitate, and institutionalize learning within a given society. The presence of structured opportunities for trial and error, such as apprenticeship, guided practice, or material affordances that encourage exploration, suggests that even individual learning often operates within a more complex social learning environment. Already according to the definitions above, culture cannot exist without ways to acquire information from others.

Several forms of social learning have been distinguished conceptually and inquired empirically, ranging from stimulus and local enhancement, observational conditioning and affordance learning, up to sophisticated forms of emulative or imitative copying [24–26]. The scope of social learning in animals is wide, including taxa as different as primates, cetaceans, fish, songbirds and even insects [27]. Contexts in which social learning has been documented in animals encompass foraging and hunting strategies, prey selection, predator recognition, breeding or egg-laying locations, migratory routes, tool use, vocal dialects and social customs [3]. The variety of social learning forms, taxa and contexts suggest that social learning is less species-specific than it might be claimed at first sight, and that emergence and/or employment of different social learning forms may be considerably contingent upon context rather than depending on phylogenetically based cognitive architectures.

Moreover, recent studies – spurred by a reevaluation of Roger’s paradox [28] and supported by experimental research, empirical findings, and mathematical models [22,23,29–31] – have emphasized the variability and flexibility of social learning at individual, intra-population, and inter-population levels. Roger’s paradox illustrates a fundamental challenge in cultural evolution: an overreliance on social learning risks perpetuating outdated or maladaptive behaviors, whereas excessive asocial learning is inefficient and costly. This research has led to the development of the concept of “social learning strategies” (SLSs), which provide a framework for understanding how organisms navigate the trade-offs highlighted by Roger’s paradox. SLSs offer solutions by allowing organisms to combine asocial and social learning in adaptive ways, either simultaneously or sequentially [32]. They also encompass the biases and heuristics that influence decisions about what, when, and whom to copy [33]. By enabling flexible and context-dependent learning, SLSs help balance innovation and transmission.

This research field provides two key insights relevant to understanding cultural transmission in human evolution. First, employing SLSs requires individuals to monitor both their own behavior and that of others, assessing the cost-effectiveness of strategies and the outcomes of observed behaviors [34]. Second, the repertoire of social learning strategies in individuals and populations can evolve over time without necessitating genetic or cognitive changes. This behavioral flexibility, observed across the animal kingdom, suggests that the evolution of social learning strategies occurs also at the cultural and behavioral levels rather than being driven solely by genetic evolution [35].

Teaching, as a complex behavioral form of social transmission, is often credited as a key for human expanded cumulative cultural evolution [36–38]. The debate about the scope of teaching, not only in the animal kingdom but also across human societies, is influenced by the many proposed definitions of teaching itself. If it is intended close to “schooling”, or as a quasi-formal procedure often called “direct active teaching” (where teacher and novice are aware that information transfer is occurring, and where knowledge is transmitted with overt communication or even verbally), then it is not only absent in non-human animals, but may also be rare (or completely lacking) in some contemporary traditional societies [36,39–42]. It has been shown, for example, that horizontal transmission between young members of a human group plays a relevant role in cultural transmission via imitation and “impregnation” and without involving formal teaching [43–45]. If, instead, teaching is understood in more “functionalist” terms as behavior put in practice by an organism *to facilitate learning* in conspecifics, then it is widespread – possibly ubiquitous – in human societies [46], and has also been documented in several non-human species of insects, birds, mammals, and primates [47 for review].

Understood in this latter way, teaching can by articulated in different forms [46]. Teaching by social tolerance occurs when an expert individual tolerates interference by a naïve one in performing some task. Teaching by opportunity provisioning occurs when an expert lends occasions for a pupil or novice to practice some relevant activity. In teaching by stimulus or local enhancement the expert calls novice’s attention to specific stimuli or locations in the environment which otherwise would go unnoticed by the novice. Teaching by evaluative feedback occurs when an expert provides feedback about the positive or negative consequences of the pupil’s behavior. Finally, direct active teaching “is characterized by (1) manifestation of relevant information by the teacher to the pupil and (2) interpretation of this manifestation in terms of knowledge content by the pupil. It differs from other teaching adaptations in that it requires some shared background knowledge as well as a means of direct communication, so that the teacher can identify and communicate the relevant information to the pupil” [46, p. 8]. None of these teaching forms necessarily require articulate language to be implemented, but all (with the partial exception of teaching by social tolerance) are significantly enhanced by the use of a verbal articulate language. From this standpoint, the evolution of transmission strategies and the evolution of language have likely interacted in our lineage [48].

All these forms of teaching are practiced by humans, though to varied extents across societies. Their distribution among animals does not identify any obvious “phylogenetic trend” and rather reflects the particular ecologies and social settings of the considered populations or species [49,50]. Therefore, like for social learning, recent developments suggest that also teaching have emerged and/or evolved *behaviorally*, with the most complex forms such as direct active teaching and schooling representing the tip of an iceberg sinking its body deep into hominin cultural evolution.

Our emphasis on the behavioral level is not to detract the importance of genetic evolution and ensuing changes in neural and cognitive architectures along the human lineage. Our understanding of the relationships between biology and culture is burgeoning since at least the mid-1970s thanks to research into gene-culture co-evolution [51–55]. Gene-culture co-evolution highlights cases in which some cultural practices (e.g., dairy farming or social preference for thin dark hairs) affect the distribution of gene variants (alleles) within a population. The relevance of gene-culture co-evolution for human evolution has been progressively enquired [56–59] and its scope is being assessed in the animal kingdom as well [60].

By definition, gene-culture co-evolution implies gene change. Culture often has a pace of evolution faster than genes: it changes also intra-generationally and cultural novelty can spread rapidly via social learning and teaching. Indeed, authors have sometimes proposed the notion of “culture-driven evolution” [61–64] to stress that in gene-culture co-evolutionary processes the first move often is on culture’s side. The phenotypic and behavioral level is more and more regarded as a primary cause of evolution, whereas genes are in some cases considered as “followers” in the process [65–69]. Accordingly, gene-culture co-evolution focuses on the *consequences* of certain cultural traits on genetic evolution. And such cultural traits may well emerge behaviorally and stabilize via social learning and teaching.

Hence, many aspects of cultural evolution can and should be studied independently of their genetic consequences. We argue that this is particularly true for the evolution of social learning, teaching, and transmission strategies – topics about which archaeology should play a central role [70–72].

Our knowledge of prehistoric cultures is expanding considerably. Findings and discoveries accumulate; the geographical and temporal patterns of several cultural traits are progressively ascertained; analysis of archaeological remains gets more and more accurate; experimental archaeology unveils arrays of details on the production and use of ancient material cultural items; ethnography allows insightful comparative analysis between archaeological findings and the material culture of contemporary traditional societies. These advancements are opening interesting windows into the *behavior* of our ancestors, allowing to reconstruct in detail how ancient artefacts were produced, how they were used and, to some extent, the social and behavioral requirements necessary for transmitting such knowledge.

By building on these advancements, archaeologists model the evolution of cultural traits exploring their implications for the evolution of cumulative culture as such. Attempts in this direction have been recently made, e.g., for the evolution of bone technology [73], the decoration of the human body [74], the evolution of sewing technology and clothing [75], the production and use of artificial memory systems [76], and ochre pigment use [77,78].

Such developments, along with the relevance attributed to social learning in cultural evolution, have prompted archaeologists to enquiry into the cognitive and neural requirements ensuring the cumulative evolution of cultural traits, to the point of proposing the fields of “cognitive archaeology” [79–81] and “neuro-archaeology” [82–84]. However, the general stance is to inquiry into the factors that have conditioned this process, and the cognitive faculties and/or neural substrates required or involved in human cumulative cultural evolution, by focusing on single specific cultural traits or trajectories. A recent debate in *Antiquity* has further highlighted the complexity and multidimensional nature of cultural transmission and innovation. Bentley and O’Brien’s [85] exploration of cultural traditions and innovations prompted varied responses, emphasizing different facets of human cultural evolution. Tim Ingold [86] criticized the perceived reductionism in their framework, advocating instead for a more imaginative and holistic approach to understanding cultural processes. Catherine Frieman [87] underscored the diversity and creativity of the past, challenging the notion of linear or deterministic cultural progress. Anna Marie Prentiss [88] highlighted the role of human agency and intentionality in shaping cultural lineages, while A.M. Pollard [89] focused on the interplay between cultural inheritance and technological evolution. Bentley and O’Brien’s [90] final response sought to find common ground, stressing the need for integrative perspectives that balance tradition and innovation.

This debate underscores the importance of approaching cultural transmission as a complex, context-dependent process shaped by diverse factors, including social learning, environmental pressures, innovation pathways and gene-culture coevolution. On this background, we aim to contribute to these discussions by providing a detailed, empirically grounded scenario for the evolution of transmission strategies and its pace in the Paleolithic.

What is missing, and badly needed in our view, is a timeline for the evolution of transmission strategies anchored to the archaeological evidence allowing for the identification of the main trends leading to the present capacity for cumulative culture. If adequately pursued and developed in the future, this effort would be able to bridge the gap between us, past hominins, and our closest extant species. Finally, it would complement the gene-culture co-evolution perspective on the evolution of the human lineage deepening our understanding of the “cultural side of the coin”. The research presented here explores the evolution of transmission strategies in the human lineage, focusing on their development at the behavioral level. Using archaeological evidence, we investigate how specific transmission strategies emerged and became critical to the cumulative cultural evolution observed in modern humans.

We argue that understanding the evolution of cultural transmission requires analyzing the spatial, temporal, and social dimensions of concrete interactions through which information flows between practitioners/experts and learners/pupils [71]. These dimensions provide a comprehensive framework for exploring how cultural traits are transmitted, maintained, and refined over time, offering insights into the mechanisms driving cumulative cultural evolution. The spatial dimension highlights the varying degrees of proximity and intentionality involved in learning, from distant observation to direct, intentional guidance – a progression that reflects increasing investment in teaching and its role in fostering complex cultural traits. The temporal dimension addresses the frequency, sequencing, and modularity of learning events, capturing how different cultural traits require varying levels of repetition or structured instruction, which are crucial for understanding the development of multistep technologies or symbolic behaviors. The social dimension examines the diversity of pathways through which knowledge is transmitted across generations and within communities, emphasizing the dynamic interplay of vertical, horizontal, and oblique transmission modes in shaping cultural evolution. By investigating these modes, we can better understand the conditions under which cultural traits emerged, stabilized, and entered cumulative evolution. This approach not only clarifies the behavioral mechanisms underlying cultural transmission but also aligns with archaeological evidence, offering a nuanced perspective on how hominin transmission strategies evolved to support increasingly complex cultural repertoires. Assessing the demands of learning prehistoric technologies should require, in principle, careful consideration of prior knowledge and skill sets. We acknowledge that modern archaeologists—including ourselves—are not native practitioners of prehistoric technologies, and that our evaluations of difficulty are shaped by the cognitive and technological environment in which we operate. What is perceived as challenging depends on the learner’s prior experience, making it difficult to ascertain past learning demands directly. To mitigate this potential bias, our assessments of learning complexity are not based on isolated professional judgment alone but are informed by multiple lines of evidence: (i) experimental archaeology, which provides empirical insights into skill acquisition and transmission; (ii) ethnographic and ethnohistorical data, offering analogies from non-industrial societies; and (iii) comparative technological studies, which highlight cross-cultural patterns in skill transmission. By integrating these sources, we aim to approach prehistoric learning processes with a more nuanced and empirically grounded perspective.

The dataset we created to meet these challenges (see *Methods*) and the performed analysis provide an empirically based scenario for the evolution of the strategies used by members of our lineage to transmit cultural traits over the past 3.3My. They show identifiable trends in the evolution of cultural transmission, and unveil a co-evolutionary dynamic between the emergence and accumulation of novel cultural traits and the complexification of transmission strategies. As discussed below, the results also have implications for the evolution of language precursors and for the origin of modern articulate language.

## Materials and methods

### Considered cultural traits

We have created a database comprising 103 cultural traits considered to be key innovations emerging in the Paleolithic (Table 1). These traits are grouped into 17 general categories, each containing a variable number of distinct traits (ranging from 2 to 21). The selection of these cultural traits was guided by the following criteria: (1) coverage of the full range and variability of cultural innovations in the Paleolithic; (2) availability of direct or circumstantial archaeological evidence; (3) presumed relevance of the trait in human niche expansion; and (4) availability of ethnographic and/or experimental data relevant to their cultural transmission. The database entry *lines* correspond to the cultural traits considered in this study, as reported in Table 1 (see also S1 Table for more details). Table 1 provides the age of the first consolidated appearance for each trait and its attribution to a time period used in our analysis (see below “Dating and Time Periods” section). S1 Table includes brief descriptions of each cultural trait and relevant references for the age of their first consolidated appearance.

**Table 1:** Cultural Traits. This Table includes the 103 cultural traits considered in this study, divided into categories. For each cultural trait the table provides the abbreviation, the age of first consolidate appearance, and the appropriate time period for statistical analysis. S1 Table adds, for each trait, a brief definition and key references for the date of first consolidate appearance in the archaeological record.

Category	Cultural trait	Trait Label	Age of first appearance	Time period
**Lithic technology**	Stone Hammer	LIstoneHammer	3.300.000	2.8-3.3My
	Mode 1 Oldowan	LIMode1	2.600.000	2-2.8My
	Mode 1 Lokalalei	LIMode1Loka	2.300.000	2-2.8My
	Acheulean	Liacheulean	1.700.000	600−2My
	Discoidal knapping	Lidiscoidal	1.000.000	600−2My
	Levallois preferential flaking (flake)	LIlevalloisFlake	400.000	300-400ky
	Levallois preferential flaking (point)	LIlevalloisPoint	400.000	300-400ky
	Levallois recurrent flaking	LIlevalloisRecFlake	400.000	300-400ky
	Bipolar knapping	Libopolar	3.300.000	2.8-3.3My
	Prismatic blade production	LIprismaticBlade	330.000	300-400ky
	Clactonian knapping	LIclactonFlake	400.000	300-400ky
	Clactonian retouching	LIclactonRetNotch	400.000	300-400ky
	Microblade production with soft hammer	LIMicroBladeSoft	42.000	0-50ky
	Pressure Blade production	LIPressureKnap	44.000	0-50ky
	Quina retouching	LIQuinaRetouch	420.000	400-600ky
	Soft hammer retouching	LIsoftRetouch	500.000	400-600ky
	Hard hammer retouching	LIHardRetouch	2.300.000	2-2.8My
	Pressure retouching	LIpressRetouch	73.000	50-100ky
	Microliths (shaped)	LIshapedMicroliths	65.000	50-100ky
	Lower Grindstone	LIlowerGrind	780.000	600−2My
	Lower and Upper Grindstone	LILowerUpperGrind	70.000	50-100ky
	Complex bifacial shaping	LIcomplBifaShape	90.000	50-100ky
**Mastic**	Without pyrotechnology	MASTICsimple	180.000	100-200ky
	With pyrotechnology	MASTICPyro	180.000	100-200ky
	With addiction of other substances	MASTICcompound	44.000	0-50ky
	With pigment	MASTICpigment	65.000	50-100ky
**Hafting**	Simple hafting	HAFTsimple	500.000	400-600ky
	Complex (multi-element) hafting	HAFTmultiElement	200.000	100-200ky
**Simple bone tools**	Used bones	BOTOOLused	2.400.000	2-2.8My
	Retouchers	BOTOOLRetoucher	600.000	400-600ky
	Knapped bones	BOTOOLknapped	1.500.000	600−2My
**Formal bone tools**	Barbed points	BOTOOLFObarb	95.000	50-100ky
	Harpoons	BOTOOLFOharp	18.000	0-50ky
	Bone spear points	BOTOOLFOspear	75.000	50-100ky
	Bone awls	BOTOOLFOawl	80.000	50-100ky
	Bone arrowpoints	BOTOOFOLarrow	70.000	50-100ky
	Wedges	BOTOOLFOwedge	65.000	50-100ky
	Handles	BOTOOLFOhandle	35.000	0-50ky
	Eyed needles	BOTOOLFOneelds	45.000	0-50ky
**Ornaments**	Natural items	BEADnatural	140.000	100-200ky
	Artifically perforated or grooved items	BEADperf	80.000	50-100ky
	Artificially colored items	BEADcolour	140.000	100-200ky
	Items blackened by heating	BEADblack	80.000	50-100ky
	Fully shaped beads	BEADshaped	50.000	0-50ky
	Complex beadwork (using different kinds of items)	BEADmultiple	70.000	50-100ky
	Modified rare or exotic items (finished objects; focus on its value)	BEADvalue	45.000	0-50ky
**Pigments**	Selected raw material	PIGselected	500.000	400-600ky
	Thermal treatment	PIGheated	110.000	100-200ky
	Multiple processing strategies	PIGmultiProcess	100.000	50-100ky
	Determination of powder texture (granulometry)	PIGMultiGrain	80.000	50-100ky
	Use of a binder	PIGbinder	65.000	50-100ky
**Representations**	Incising Abstract Single technique	ARTincAbstrSingleTech	110.000	100-200ky
	Incising Abstract Multiple technique	ARTincAbstmultipleTech	75.000	50-100ky
	Painting Abstract Monochromatic	ARTpaintAbstrmonochrome	64.000	50-100ky
	Painting Abstract Polychromatic	ARTpolychrome	40.000	0-50ky
	Incising Depictional Single technique	ARTincDepicSingleTech	40.000	0-50ky
	Incising Depictional Multiple technique	ARTincDepicMultTech	40.000	0-50ky
	Painting Depictional Monochromatic	ARTPaintDepicMonochrome	45.000	0-50ky
	Painting Depictional Polychromatic	ARTPaintDepicPolyChrome	35.000	0-50ky
	Carving Abstract	ARTcarvingAbstr	40.000	0-50ky
	Carving Depictional	ARTcarvingDepic	40.000	0-50ky
	Multi-mode (e.g., pianting and carving)	ARTmultimode	40.000	0-50ky
**Mortuary practices**	Curation	MORTcuration	320.000	300-400ky
	Morbidity	MORTmorb	3.300.000	2.8-3.3My
	Cronos compulsion	MORTcronos	800.000	600−2My
	Abandonment	MORTabandon	3.300.000	2.8-3.3My
	Structured abandonment	MORTstructAbandonment	3.300.000	2.8-3.3My
	Funerary caching	MORTfuneraryCach	430.000	400-600ky
	Cairn covering	MORTcairnCover	55.000	50-100ky
	Formal burial or inhumation	MORTburial	120.000	100-200ky
	Place of multiple burial	MORTmultiburial	65.000	50-100ky
	Cemetery	MORTcemetery	11.000	0-50ky
	Detachment	MORTdetach	450.000	400-600ky
	Commemoration	MORtcommemo	40.000	0-50ky
**Artificial Memory Systems**	Simple finger counting	AMSfingCount	30.000	0-50ky
	Complex finger counting	AMScomplexFingCou	7.000	0-50ky
	Verbal counting	AMSverbalCou	70.000	50-100ky
	Notation by accumulation	AMSnotaAccum	44.000	0-50ky
	Notation based on marks morphology, spatial distribution and accumulation	AMSMorphSpat	40.000	0-50ky
	Number symbols	AMSnumberSy	6.000	0-50ky
**Wooden artifacts**	Modified wooden tools (chimps brush or spade)	WOODmodif	3.300.000	2.8-3.3My
	Tool-modified wooden tools	WOODshaped	400.000	300-400ky
	Bows and arrows	WOODbowArrow	65.000	50-100ky
	Handles	WOODhandle	400.000	300-400ky
**Poison**	Single-ingredient poison	POIsingle	78.000	50-100ky
	Compounded poison	POIcompound	44.000	0-50ky
**Pharmacy**	Simple consumption	PHAsimple	3.300.000	2.8-3.3My
	Preparation of one substance	PHAprepa	225.000	200-300ky
	Preparation and combination of differnt substances	PHAcompound	44.000	0-50ky
**Long distance exchange**	Raw material	LONGDIrawmat	300.000	200-300ky
	Special functional objects	LONDIspecialOb	280.000	200-300ky
	Exoctic goods	LONDIexotics	105.000	100-200ky
**Fire**	Fire control, striking	FIREstriking	400.000	300-400ky
	Fire control, drill	FIREdrill	400.000	300-400ky
	Pyrotechnology	FIREpyro	140.000	100-200ky
**Seafaring**	Coastal navigation	SEAcostal	870.000	600−2My
	Unintentional or opportunistic island crossing (with visible land)	SEAseeDestination	870.000	600−2My
	Early hominin crossings to non-visible landmasses	SEAnotSeeDestination	130.000	100-200My
	Ocean exploration	SEAunknownDestination	50.000	0-50ky
**Fishing technologies**	Hand catching	FISHhand	300.000	200-300ky
	Fishing traps	FISHtrap	65.000	50-100ky
	Fish hook	FISHhook	23.000	0-50ky
	Fish spearing	FISHspear	95.000	50-100ky

Our selection of traits prioritizes *manufacturing techniques* in cases where the procedural steps leading to an outcome are identifiable by archaeologists but the ultimate goal of the technique is variable or uncertain. For example, different Levallois debitage systems can result in the production of a Mousterian scraper, yet the discovery of such a scraper does not always reveal the specific debitage methods used to produce the blank subsequently retouched to produce the scraper. Moreover, the scraper itself often provides limited information about the complexity of the transmission strategies involved. By focusing on techniques in such cases, we aim to capture the transmission challenges associated with procedural complexity rather than inferred end products. Conversely, when dealing with *end products* like Acheulean bifaces, eyed bone needles, or retouched microliths intended for hafting, the emphasis shifts to their defining characteristics – such as shape, function, miniaturization, or their role as components of composite tools. These traits may have been produced through different manufacturing methods, which are only partially understood in some cases. However, their significance lies in their functionality and the cognitive, social, and technical requirements for their creation and use, which inform our understanding of transmission strategies.

We acknowledge that a degree of ambiguity is intrinsic to certain traits, reflecting the current state of archaeological knowledge. The identification and interpretation of traits – and the timing of their first consolidated appearance – are subject to ongoing refinement as new discoveries and analytical techniques emerge. If this database had been created two decades ago, it would undoubtedly have differed significantly in its composition, highlighting the dynamic nature of the field and the evolving interpretations of archaeological evidence. It is also important to recognize that the choice of traits is influenced by the history of the discipline and the ways in which archaeological research has traditionally approached the record. For instance, emphasis has often been placed on the first occurrence of technological innovations, even when subsequent research has shown these to be isolated occurrences. This reflects not only the empirical record but also the historical priorities of archaeological research.

### Dimensions and modes of cultural transmission

The database entry *columns* represent distinct modes of cultural transmission that ensure the maintenance of a cultural trait within a population. These modes are intended to capture the concrete behavioral interactions between a practitioner and a learner. A practitioner (or expert) is the individual (or individuals) who possess(es) a body of knowledge and use(s) it to perform a task. Practitioners may or may not intend to teach or facilitate the learning of their knowledge by a learner. A learner (or novice) is an individual, usually a conspecific in our framework, who either intends to learn, is intended to learn, or just learns a particular body of knowledge from a practitioner.

We consider that cultural transmission occurs in three different *dimensions* (spatial, temporal and social) in which information is transferred from practitioners to learners [71].

The spatial dimension concerns the spatial relationships between practitioner and learner during transmission events. Such relationships might be merely spatial (physical distance) or also encompass specific classes of actions or behaviors the practitioner put in practice to facilitate the novice’s learning. Modes within this dimension are:

*Distant observation* (SPAdistal). The learner observes a practitioner’s actions from a distance (not in close proximity); the practitioner does not intend to teach, might be unaware of the observing learner, or may even attempt at masking/concealing its actions.

*Disconnected proximal observation* (SPAproDis). The practitioner accepts the proximate presence of the learner but is unaware of, or uninterested in, novice’s learning.

*Conscious proximal observation* (SPAproCon). The practitioner tolerates the learner’s presence and is to an extent aware that the latter is there to acquire the knowledge necessary to perform the same task and achieve the same goal, but does nothing to facilitate learning.

*Proximate observation with practitioner intentionality* (SPAproInt). The practitioner’s actions are intentionally performed in such a way (e.g., slowed down, exaggerated, etc.) that novice’s learning is actively facilitated. Note that in this transmission mode the practitioner’s focus may be more on transmitting information than in performing a task successfully.

*Gesture molding* (SPAgeMo). The practitioner guides the learner’s actions through physical contact. This concerns cultural traits that cannot be acquired by simple observation, and require the practitioner to shape the learner’s movements physically.

*Complemented gesture molding* (SPAgeCo). The previous mode (SPAgeMo) is accompanied by positive or negative non-verbal feedback the practitioner overtly gives to the novice.

*Explanation complemented action* (SPAexpAct). The practitioner complements its actions – or the “enacting” of these actions – with overt explanations allowing the learner to understand better the actions, some of their details, their order, and/or their goals. This transmission mode might not need full-blown spoken articulate language but requires a complex-enough communication system.

*Explanation in absence of action* (SPAabsAct). Knowledge is communicated without the real execution of the actions necessary to achieve the goal. This may involve articulate language but also less complex forms of communication like gestural languages, protolanguages, etc.

The temporal dimension concerns the timing, frequency and/or order of transmission events to transmit a cultural trait to a learner. Modes within this dimension are:

*Single information transfer* (TEsingle). The necessary knowledge can be transmitted/acquired in a single transmission event; even though the novice might still need time and rehearsal to perfect it, this will not require further transmission.

*Repetitive single information transfer* (TErepetitive). The necessary knowledge needs repeated transmission events of the same kind to be successfully transmitted/acquired.

*Sequential information transfer* (TEsequent). Acquiring the knowledge necessary to perform a task entails a sequence of learning events about different action segments that must occur in a given order.

*Modular information transfer* (TEmodular). Acquiring the necessary knowledge to perform a task requires learning a number of distinct operative “modules” that can be acquired in an order different from that required to complete the whole task. In this scenario, a single operative module can be involved in the implementation of more than one cultural trait.

*Disconnected information transfer* (TEdisco). Acquiring the necessary know-how to perform a task implies learning several disconnected pieces of knowledge the utility of which will only become apparent at a future stage, when they will be integrated in order to achieve the goal. In this scenario, the learner might even be unaware of the final task/goal when acquiring (some of) the pieces of knowledge.

The social dimension concerns the social “directions” in which information flows, i.e., who learns from whom. Modes within this dimension are:

*Horizontal, vertical and oblique transmission* (SOhoVeOb). In our framework, horizontal transmission happens through related individuals of the same generation; vertical transmission goes from parents to offspring; oblique transmission occurs when the novice receives information from relatives other than parents and siblings.

*Horizontal, vertical and oblique selective transmission* (SOhoVeObSe). It is the same scenario as above but information is transmitted *selectively* – i.e., specific practitioners pass information only to selected novices (e.g., from mothers to daughters, or only to first-borns, etc.).

*Generalized hyper-oblique transmission* (SOhyObGe). Within an extended social group, information is passed on from any expert individual to any novice, disregarding genetical or social closeness and without selectivity.

*Selective generalized hyper-oblique transmission* (SOhyObSe). It is the same scenario as above but cultural traits are transmitted in a selective manner – e.g., according to social status, gender, or other criteria.

*Reciprocal generalized hyper-oblique transmission* (SOhyObReGe). This is the same scenario as *generalized hyper-oblique transmission* with the relevant addition that members of a younger generation may transmit a (novel) cultural trait to older generations.

*Reciprocal selective generalized hyper-oblique transmission* (SOhyObReSe). This is the same scenario as *selective generalized hyper-oblique transmission* with the relevant addition that selected members of a younger generation may transmit a (novel) cultural trait to specific members of the older generation.

### Database scores and assignment procedure

The database has been filled by attributing a score from 0 to 4 to each mode of cultural transmission (*columns*) for each cultural trait (*lines*) (S1 File). Scores for the transmission modes in the *spatial* and *temporal* dimensions were assigned according to the following criteria:

1. It plays no role in the transmission of any aspect of the cultural trait, or is not applicable to the case in point.
2. It allows one to understand the end-goal of the action.
3. It allows one to understand the end-goal of the action and provides elements of the necessary knowledge which however are not sufficient to reproduce the cultural trait.
4. It contributes substantially to the transmission of the cultural trait without guarantying its full acquisition.
5. It is the most important mode to transmit the cultural trait and ensures its full reproduction and transmission to new generations of learners.

The highest score (= 4) was attributed, in each of these two dimensions, to the transmission mode considered to be the *least complex but sufficient* to ensure full conveyance of the cultural trait. The designation of the “least complex but sufficient” transmission mode follows an established principle in cognitive science and anthropology, which prioritizes efficiency in cultural transmission. This principle assumes that individuals tend to adopt the simplest effective method to ensure reliable transmission, minimizing unnecessary complexity [46,48]. For each cultural trait, identifying the least complex but sufficient mode involved evaluating its transmission requirements against this heuristic while cross-referencing evidence from expert consultations and literature. By incorporating feedback from domain experts and aligning with established frameworks, we ensured that our scoring reflects a synthesis of empirical data and theoretical understanding. The other modes of cultural transmission where assigned scores from 0 to 3. For each cultural trait, a single 4 was assigned within each dimension (spatial and temporal), whereas scores between 0 and 3 may have been attributed more than once. The logic behind this scoring system is that cultural traits are often transmitted with contribution by several transmission strategies in each of the considered dimensions.

Scores for the *social* dimension reflect the level of likelihood with which the social transmission mode may have been employed to pass the trait on, or the relevance of the considered social mode in transmitting the trait. In the social dimension, each score (score = 4 included) might have been assigned more than once for each trait.

The scores were assigned following a three-step procedure (see details below). First, the two authors jointly have assigned the scores for each cultural trait. Then they have asked experts in each of the considered traits to assign their scores. In some cases, experts did not assign numerical scores but provided their assessment by answering relevant questions. Finally, the authors have refined the original scores on the basis of the experts’ feedbacks.

In the first step, to assign scores the authors took into account:

- data from the archaeological literature on the *chaînes opératoires* involved in implementing the cultural traits;
- insights from the ethnographic literature on how traits included in the database are transmitted in traditional societies;
- observations from the reproduction of the cultural traits by modern experimenters and the transmission of this traits to present-day learners;
- findings from ethological literature on how a cultural trait considered in the database is implemented and transmitted in other animal species, particularly primates;
- first-hand observations and experimental reproduction of cultural traits by one of the authors (FD).

After synthesizing the above information, each transmission mode, in each dimension, was assessed to determine its probable contribution in the transmission of each cultural trait. First, the *least complex but sufficient* transmission mode was singled out and assigned a score of 4. The transmission modes regarded to play no role in the transmission were assigned a score of 0. The other transmission modes were assigned a score from 1 to 3 following the criteria explained above. Note that a score of 1–3 may have been assigned to a certain transmission mode either (i) as it appears to be insufficient to transmit the trait or (ii) as it may be effective but exceeds the *least complex but sufficient* transmission mode for that trait (i.e., it is more than the trait transmission actually requires).

In the second step, archaeologists and cultural anthropologists (listed in the Acknowledgments section) with a demonstrated expertise in one or more cultural traits within a given category were contacted. They were introduced to the dimensions and modes of cultural transmission, and to the criteria for assigning scores. Depending on their availability, some experts were directly interviewed and asked to assign their scores for the cultural traits within their expertise. The interviewed experts were not aware of the scores assigned by the authors beforehand. In this case, scores given by experts were recorded on an Excel spreadsheet by the authors in the experts’ presence. For those experts who were not available for direct interviews, contact was made by e-mail. After being introduced to the project goals as well as the dimensions and modes of cultural transmission under consideration, these experts provided their responses by indicating the weight they would attribute to different modes of cultural transmission for the perpetuation of the relevant cultural traits. In this case, the emails with experts’ answers were archived by the authors in a text file. All experts who have given their opinions, as listed in the Acknowledgment section of this article, reviewed and signed a consent form also stating the purpose for which their opinions were asked. For each cultural trait, at least one and no more than three experts’ advices were received. Although it was not feasible, following the above procedure, to individually cite every source used to assess the transmission modes for all 103 cultural traits, the scoring process drew extensively on the collective expertise of the authors and feedback from consulted specialists.

In the third step, scores provided by the directly interviewed experts were compared to those assigned by the authors and discrepancies were assessed. The answers received by scholars interviewed via email were reviewed for the same purpose. This overall process helped identify aspects of the cultural traits transmission that were not adequately addressed by the authors in the first step. Therefore, the third step resulted either in no change in the original scores assigned by the authors, acceptance of the scores suggested by the experts, or assignment of a new score by the authors. Our dataset comprises 1,957 datapoints (103 cultural traits times 19 variables). After the third step we have modified scores in 234 datapoints (11,96%) of which: 147 (7,51%) has been changed by +/- 1, 69 (3,53%) by +/- 2, 15 (0,77%) by +/- 3, and in only 3 cases (0,15%) by +/- 4 (see S2 Table). These data indicate good agreement between the scores originally assigned by the authors and those proposed by the experts, with less than 1% displaying a discrepancy of +/- 3 or 4. The final dataset (S1 File) reflects scores that integrate expert feedback.

### Dating and time periods

The database also includes the age of the first consolidated appearance of each cultural trait. By “age of consolidated appearance” we mean the time at which a trait is consistently observed in the archaeological record, and not the time of early sporadic and often controversial instances of a trait. This choice is motivated not only by the need to refer to sound archaeological data, but also as enquiring cultural transmission strategies entails consideration of cultural traits effectively transmitted over many generations.

In addition, we also partitioned the cultural traits in 9 time periods (0–50ky, 50–100ky, 100–200ky, 200–300ky, 300–400ky, 400–600ky, 600ky-2My, 2–2.8My, 2.8–3.3My) according to their date of first consolidated appearance. Table 1 reports both the date of first consolidated appearance and the assigned time period for each cultural trait; S1 Table also indicates, for each cultural trait, key references for dating. When information available in the literature about the dating of a cultural trait was ambiguous, we assigned the date corresponding to the best documented consolidated presence of the trait in the archaeological record.

The temporal divisions used in this analysis were determined by three criteria: (1) ensuring sufficient trait density in each interval to allow for robust statistical analysis, (2) aligning with widely accepted chronological boundaries marking key transitions in human cognitive, technological, and cultural evolution, and (3) providing transparency by presenting raw trait-specific results alongside aggregated trends. For instance, the 50,000-year boundary corresponds to a period of rapid innovation in symbolic behavior, while the 600,000-year mark reflects technological advances and the Acheulean expansion. These boundaries align with established literature [14,15]. Alternative temporal frameworks, including evenly spaced intervals, were tested and yielded consistent results, further validating our approach. Importantly, inferences about the cultural transmission of each trait are not aggregated into time periods. Instead, results are provided for each cultural trait individually to ensure clarity and specificity. The time periods are employed only as analytical units for exploratory analyses, allowing for the detection of broader trends while preserving the resolution of trait-specific findings. Moreover, raw data for each trait are included in figures, enabling readers to assess trends independently of the time periods.

### Preservation impact

To evaluate the impact of preservation biases and detectability on the temporal reconstruction of cultural traits, we assigned scores (S3 Table) to each trait based on four key factors:

*Conservation* in the Archaeological Record – This criterion assesses the ease with which a cultural trait is preserved over time, considering taphonomic processes and material durability. Traits that are highly resistant to degradation (e.g., lithic artifacts) receive a score of 0 (easily preserved), while those that are highly perishable (e.g., organic materials, pigments) receive a score of 3 (not easily preserved).

*Continuity* in the Archaeological Record – This criterion evaluates whether a cultural trait is consistently observed after its first documented occurrence. Traits that appear continuously over time receive a score of 0 (highly continuous), while those that are sporadic or show long gaps between occurrences receive a score of 3 (highly discontinuous).

*Number of Occurrences* – This factor captures the amount of documented evidence supporting the presence of a cultural trait. Traits that are well-represented in the archaeological record, appearing in many sites or contexts, receive a score of 0 (abundant occurrences), while those known from only a few isolated cases receive a score of 3 (few occurrences).

*Detectability* – This criterion assesses how easily a cultural trait can be recognized in the archaeological record. Traits that are immediately visible and require minimal analysis (e.g., large stone tools) receive a score of 0 (easily detectable), while those that require specialized techniques such as microscopic analysis or chemical residue studies (e.g., use-wear on tools, ochre processing) receive a score of 3 (hardly detectable).

### Statistical analysis on the database

To test whether preservation biases systematically affect the temporal order of cultural traits, we performed a Spearman’s rank correlation analysis between the first documented occurrence of cultural traits and the four preservation bias factors (S2 File). This non-parametric test was selected due to the ordinal nature of preservation bias scores and the potential for non-linear relationships. Additionally, we conducted a multiple linear regression analysis to evaluate whether preservation bias factors significantly predict the first occurrence age of cultural traits. The regression model included First Appearance Age as the dependent variable and Conservation, Continuity, Number of Occurrences, and Detectability as independent variables.

To test the robustness of our chronological framework, we applied a random perturbation model to simulate the effect of dating uncertainties. We introduced stochastic perturbations as follows: 33% of cultural traits were randomly shifted by ±10% (to represent minor dating uncertainties); 33% of cultural traits were randomly shifted by ±20% (to simulate larger dating uncertainties); 33% of cultural traits remained unchanged (to reflect traits with more precise dating). This perturbation approach ensures a balanced distribution of uncertainty and accounts for real-world variability in archaeological dating precision. After applying these perturbations, we computed the Spearman rank correlation between the original and perturbed rankings to assess the stability of trait ordering under dating uncertainty. (S2 File).

To evaluate the robustness of our predefined time periods (0–50ky, 50–100ky, 100–200ky, 200–300ky, 300–400ky, 400–600ky, 600ky-2My, 2–2.8My, 2.8–3.3My), we performed a sensitivity analysis by adjusting their boundaries by ±10% and ±20% and reassigning cultural traits accordingly (S2 File). The goal was to assess whether moderate shifts in time period divisions would significantly alter the classification of traits, potentially affecting the validity of our framework. For each predefined time period, we calculated adjusted lower and upper boundaries using the following factors: ± 10% shift, i.e., each boundary was adjusted by multiplying by 0.9 (lower bound) and 1.1 (upper bound); ± 20% shift, i.e., each boundary was adjusted by multiplying by 0.8 (lower bound) and 1.2 (upper bound). We then reassigned each cultural trait to the nearest adjusted time period and calculated the proportion of traits that remained in their original category. If most traits remained within the same classification, this would indicate that the framework is stable to moderate boundary shifts. Additionally, we conducted Chi-square tests for independence to statistically evaluate whether the classification of cultural traits into the original vs. shifted time periods was significantly structured. The null hypothesis assumed that cultural trait assignments would be independent of time period definitions, while significant results would indicate that the original classification retains structure despite moderate shifts in boundaries.

**Fig 1:**
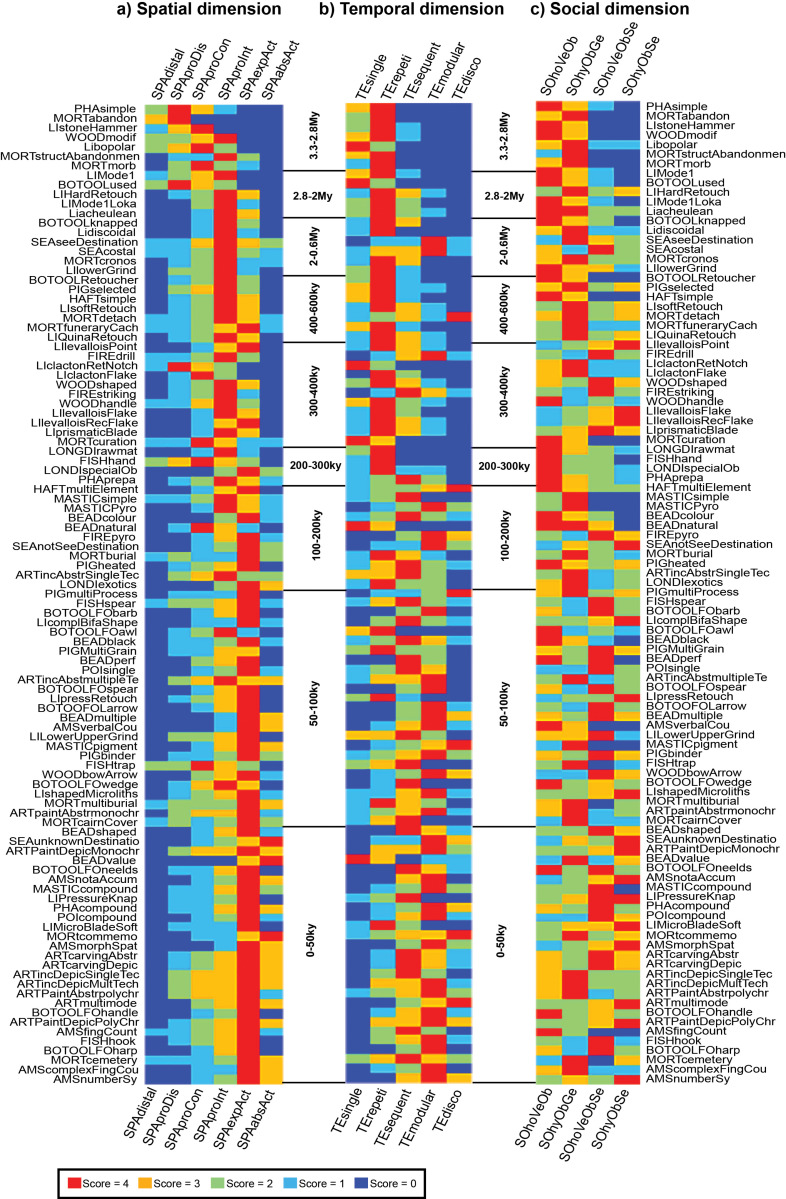
Relevance and timeline of transmission modes for cultural traits. Matrix plot visually representing with a color-code the contribution of different modes of cultural transmission (see *Methods* for the definition of transmission modes and labels) in the maintenance of 103 cultural traits that emerged during the Paleolithic (see also Table 1 and S1 Table). The cultural traits are organized chronologically based on the time of their first consolidated appearance in the archaeological record. The gesture molding modes in the spatial dimension and the reciprocal modes in the social dimension are not shown. Short definitions of cultural traits in alphabetic order by label (for longer definitions see S1 Table): AMScomplexFingCou: Complex finger counting; AMSfingCount: Simple finger counting; AMSMorphSpat: Notation based on marks morphology, spatial distribution, and accumulation; AMSnotaAccum: Notation through the accumulation of marks; AMSnumberSy: Use of number symbols; AMSverbalCou: Verbal counting; ARTcarvingAbstr: Abstract carvings; ARTcarvingDepic: Depictional carvings; ARTincAbstmultipleTech: Abstract incisions using multiple techniques; ARTincAbstrSingleTech: Abstract incisions using a single technique; ARTincDepicMultTech: Depictional incisions using multiple techniques; ARTincDepicSingleTech: Depictional incisions using a single technique; ARTmultimode: Art production involving multiple modes (e.g., painting and carving); ARTpaintAbstrmonochrome: Abstract monochromatic paintings; ARTPaintDepicMonochrome: Depictional monochromatic paintings; ARTPaintDepicPolyChrome: Depictional polychromatic paintings; ARTpolychrome: Abstract polychromatic paintings; BEADblack: Ornaments blackened by heating; BEADcolour: Artificially colored ornaments; BEADmultiple: Complex beadwork combining different kinds of items; BEADnatural: Natural items used as ornaments; BEADperf: Ornaments artificially perforated or grooved; BEADshaped: Fully shaped beads; BEADvalue: Modified rare or exotic ornaments, emphasizing their value; BOTOOFOLarrow: Bone arrow points; BOTOOLFOawl: Bone awls; BOTOOLFObarb: Barbed bone points; BOTOOLFOhandle: Bone handles; BOTOOLFOharp: Bone harpoons; BOTOOLFOneelds: Eyed bone needles; BOTOOLFOspear: Bone spear points; BOTOOLFOwedge: Bone wedges; BOTOOLknapped: Knapped bone tools; BOTOOLRetoucher: Bone retouchers or hammers; BOTOOLused: Used bones; FIREdrill: Fire control using a drill; FIREpyro: Pyrotechnology; FIREstriking: Fire control using striking techniques; FISHhand: Hand-catching fish; FISHhook: Fish hooks; FISHspear: Fish spearing; FISHtrap: Fishing traps; HAFTmultiElement: Complex multi-element hafting; HAFTsimple: Simple hafting; Liacheulean: Acheulean tools; Libopolar: Bipolar knapping; LIclactonFlake: Clactonian knapping (flake production); LIclactonRetNotch: Clactonian retouching (notches); LIcomplBifaShape: Complex bifacial shaping; Lidiscoidal: Discoidal knapping; LIHardRetouch: Hard hammer retouching; LIlevalloisFlake: Levallois preferential flaking (flake production); LIlevalloisPoint: Levallois preferential flaking (point production); LIlevalloisRecFlake: Levallois recurrent flaking; LIlowerGrind: Lower grindstone; LILowerUpperGrind: Lower and upper grindstones; LIMicroBladeSoft: Microblade production with a soft hammer; LIMode1: Mode 1 (Oldowan); LIMode1Loka: Mode 1 (Lokalalei); LIpressRetouch: Pressure retouching; LIPressureKnap: Pressure blade production; LIprismaticBlade: Prismatic blade production; LIQuinaRetouch: Quina retouching; LIshapedMicroliths: Shaped microliths; LIsoftRetouch: Soft hammer retouching; LIstoneHammer: Stone hammers; LONDIexotics: Long-distance exchange of exotic goods; LONDIspecialOb: Long-distance exchange of special functional objects; LONGDIrawmat: Long-distance exchange of raw materials; MASTICcompound: Mastics made by combining substances; MASTICpigment: Mastics made with pigment; MASTICPyro: Mastics made with pyrotechnology; MASTICsimple: Mastics made without pyrotechnology; MORTabandon: Abandonment; MORTburial: Formal burial or inhumation; MORTcairnCover: Cairn covering; MORTcemetery: Cemetery use; MORTcommemo: Commemoration; MORTcronos: Cronos compulsions (ritual timing); MORTcuration: Curation; MORTdetach: Detachment; MORTfuneraryCach: Funerary caching; MORTmorb: Morbidity practices; MORTmultiburial: Places with multiple burials; MORTstructAbandonment: Structured abandonment; PHAcompound: Combination of pharmaceutical substances; PHAprepa: Preparation of a single pharmaceutical substance; PHAsimple: Use of simple pharmaceutical substances; PIGbinder: Use of a binder in pigments; PIGheated: Thermal treatment of pigments; PIGMultiGrain: Preparation of pigments with specific textures; PIGmultiProcess: Multiple strategies for pigment preparation; PIGselected: Selected raw materials for pigment preparation; POIcompound: Compounded poisons; POIsingle: Single-ingredient poisons; SEAcostal: Coastal navigation; SEAnotSeeDestination: Early hominin crossings to non-visible landmasses; SEAseeDestination: Unintentional or opportunistic island crossing (with visible land); SEAunknownDestination: Ocean exploration; WOODbowArrow: Bows and arrows; WOODhandle: Wooden handles; WOODmodif: Modified wooden tools (e.g., chimp brushes or spades); WOODshaped: Shaped wooden tools.

Statistical analyses of the dataset containing the scores of each transmission mode in all dimensions for each cultural traits (S1 File) were performed using the Past4 software (version 1.0.4) [91] and Microsoft Excel (version 16.54). Each analysis was selected to address specific research questions related to the evolution of cultural transmission modes and to explore patterns in the dataset. To visually represent the relationships between cultural traits and variables, we generated a matrix plot (Fig 1), organizing the cultural traits chronologically based on the time of their initial consolidation. This graphical representation provides a clear overview of how cultural traits align with each variable over time, offering a foundational perspective on the data structure and its temporal progression.

**Fig 2:**
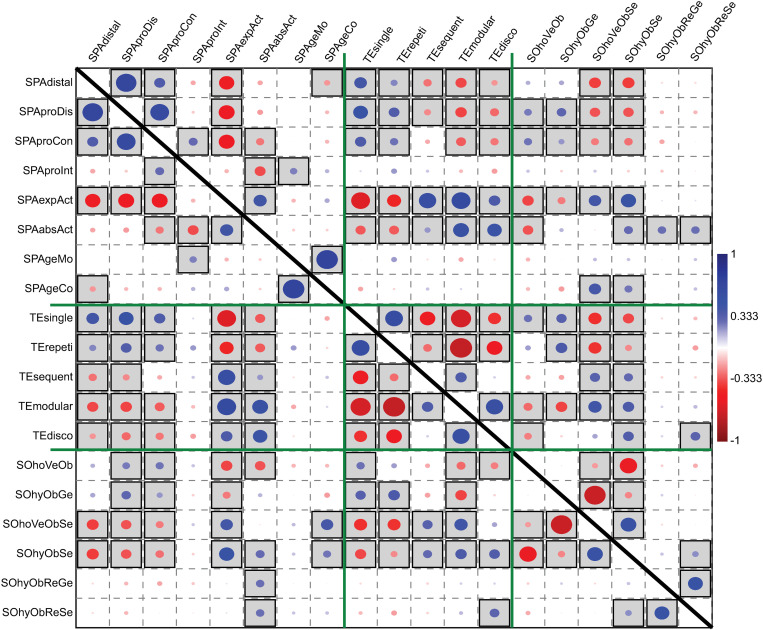
Correlations among transmission modes. Correlation analysis of the 19 modes of cultural transmission considered in this study (see *Methods* for the definitions of these variables). The size of blue and red dots indicates different degrees of positive and negative correlation, respectively (p > 0,05 boxed). Green bars help visualizing the modes of cultural transmission pertaining to the spatial, the temporal and the social dimensions. Correlation values are presented in S1 Fig.

To investigate the relationships between variables we conducted linear correlation analysis (Fig 2) using the Pearson correlation coefficient [92,93]. This method evaluates the strength and direction of linear relationships between variables, enabling us to identify significant associations that inform the co-evolutionary dynamics of transmission modes and cultural traits. The correlations help address key questions about how specific transmission strategies may have developed in tandem with cultural traits over time.

**Fig 3:**
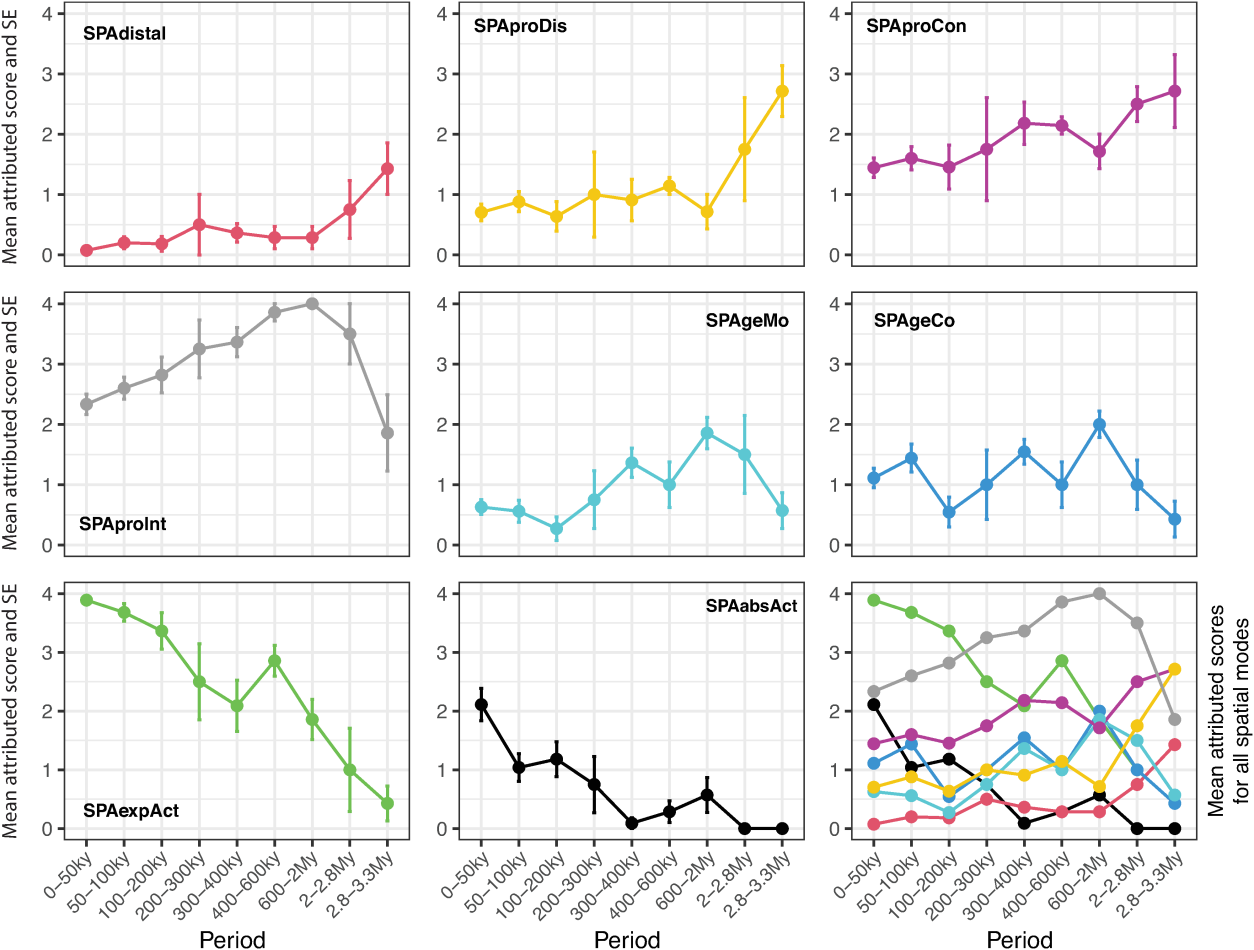
Evolution of cultural transmission modes in the spatial dimension. Variation through time in the mean value and standard error of scores attributed to the modes of cultural transmission in the spatial dimension. The spatial dimension concerns the spatial relationships between practitioner and learner during transmission events. These relationships might be merely spatial (physical distance) or also encompass specific classes of actions the practitioner put in practice to facilitate the novice’s learning (see *Methods* for the definition of transmission modes and labels). Mean values and standard errors are calculated considering all the scores given to a transmission mode within each time period (S3 File).

**Fig 4:**
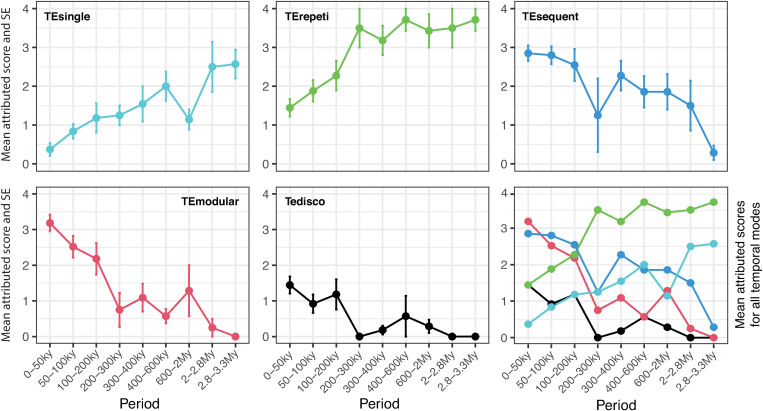
Evolution of cultural transmission modes in the temporal dimension. Variation through time in the mean value and standard error of scores attributed to the modes of cultural transmission in the temporal dimension. The temporal dimension concerns the frequency, order and/or structuration of transmission events to transmit a cultural trait to a learner (see *Methods* for the definition of transmission modes and labels). Mean values and standard errors are calculated considering all the scores attributed to a transmission mode within each time period (S3 File).

**Fig 5:**
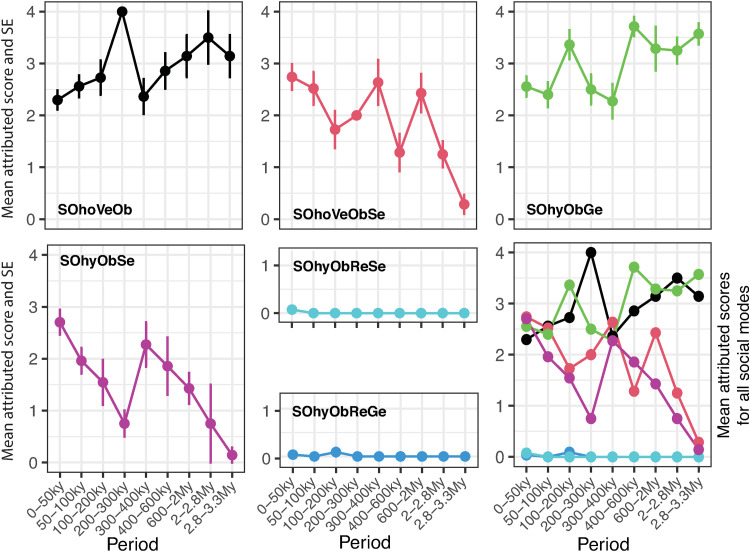
Evolution of cultural transmission modes in the social dimension. Variation through time in the mean value and standard error of scores attributed to the modes of cultural transmission in the social dimension. The social dimension concerns the social “directions” in which information flows, i.e., who learns from whom (see *Methods* for the definition of transmission modes and labels). Mean values and standard errors are calculated considering all the scores attributed to a transmission mode within each time period (S3 File).

To discern trends in the evolution of cultural transmission modes, we plotted (Figs 3–5) the mean values and standard errors of the scores assigned to each transmission mode across distinct time periods. This analysis provides a descriptive framework to visualize and compare the evolution of transmission strategies within the temporal framework of our study (see S3 File).

**Fig 6:**
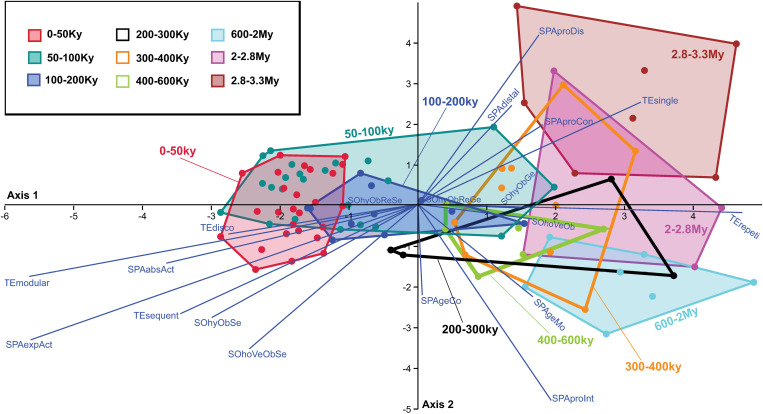
Discriminant analysis of cultural traits by transmission modes. Discriminant analysis of all (except the reciprocal social ones) the considered modes of cultural transmission (thin blue lines) and cultural traits (dots), with the latter color-coded by time periods and grouped in convex hulls (see *Methods* for the definition of transmission modes and labels). See Table 1, S1 Table and legend of Fig 1 for a definition of the cultural traits.

**Fig 7:**
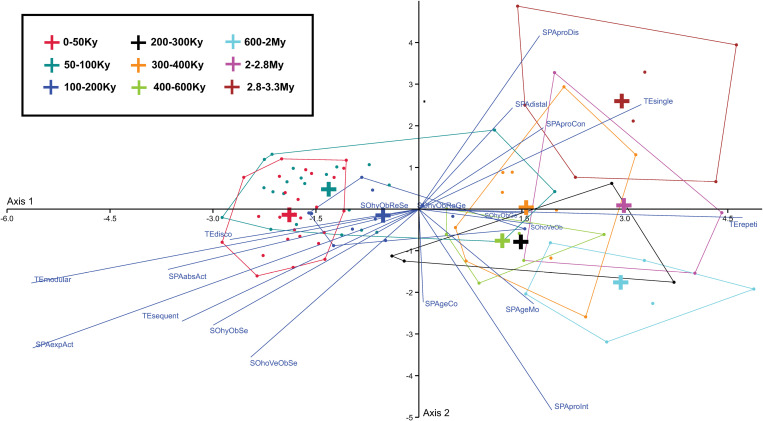
Centroids of time periods in discriminant analysis. This figure shows the centroids of the nine convex hulls sorting the considered cultural traits into time periods. The group centroid represents the mean value of the discriminant scores for a given category. The centroids are calculated by averaging the discriminant scores of all the individuals (i.e., the cultural traits) within a particular group (i.e., a time period).

We employed discriminant analysis (Figs 6, 7) to assess the discriminative power of cultural traits and transmission variables. This technique is particularly suited to our aim of identifying the factors that contribute most significantly to chronological variations in the transmission of cultural traits. Discriminant analysis allows us to evaluate whether cultural transmission modes are chronologically structured and to what extent the scores assigned to traits differentiate between time periods. This addresses the broader question of whether transmission modes evolved in response to specific cultural or environmental pressures over time.

**Fig 8:**
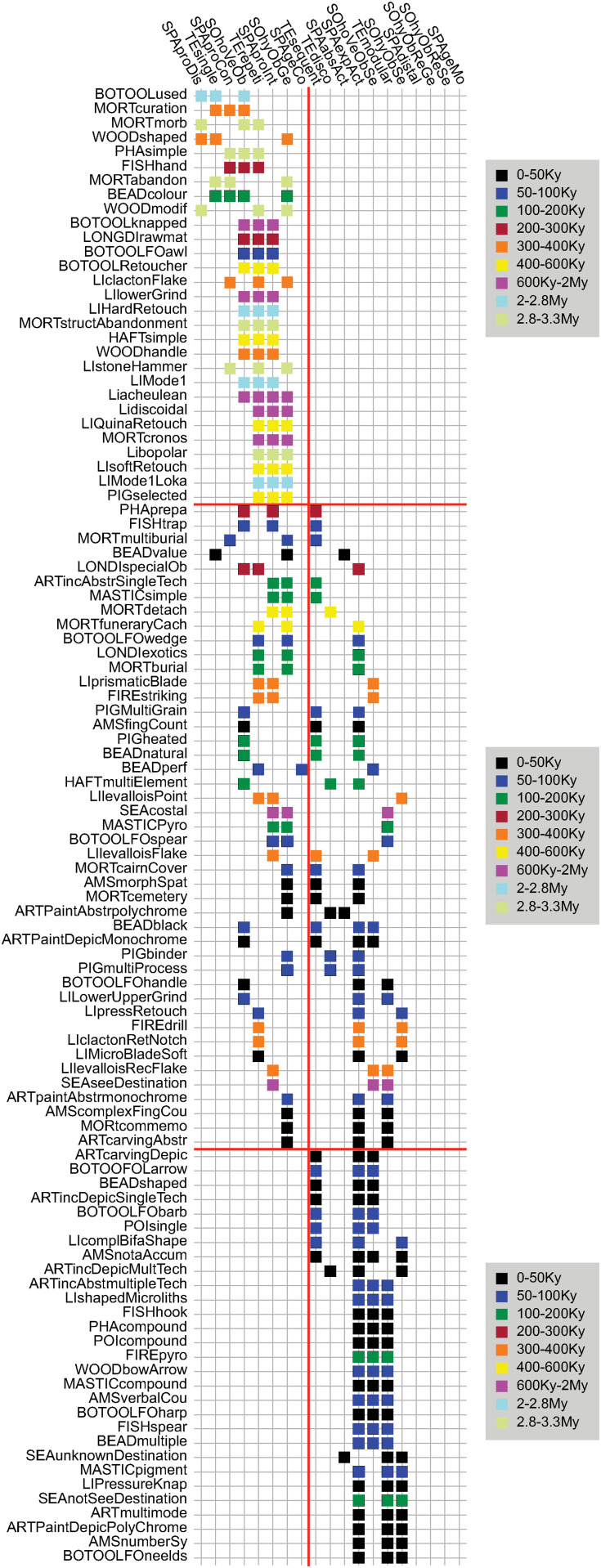
Seriation of cultural traits by transmission modes. Unconstrained seriation ordering cultural traits color-coded by time periods according to their similarity in terms of modes of cultural transmission (see *Methods* for the definition of transmission modes and labels). To obtain the seriation, the database was transformed into a presence/absence (0/1) matrix (S4 File), where original scores ranging from 0 to 3 were converted into 0, and original scores of 4 converted into 1. Thus, the figure highlights, for each dimension, which mode of cultural transmission has played the main role in the transmission of a cultural trait. See Table 1, S1 Table and legend of Fig 1 for a definition of the cultural traits.

In addition, we performed an unconstrained seriation analysis (Fig 8), following the methodology of Brower and Kile [94], to explore co-occurrence patterns of transmission modes and cultural traits. Seriation is a useful exploratory tool in archaeology to identify chronological or contextual patterns in datasets. In this analysis, we transformed the dataset into a presence/absence matrix, where scores ranging from 0 to 3 were converted to 0, and scores of 4 converted to 1 (see S4 File). This transformation simplifies the dataset for seriation, enabling us to detect patterns of association among traits and transmission modes. The use of color-coded time periods in the seriation matrix visually integrates the temporal dimension, helping to identify consistent patterns in cultural evolution.

## Results

### Robustness of cultural trait chronology and impact of preservation bias

Spearman’s rank correlation analysis (S2 File) revealed weak and statistically non-significant correlations between the first appearance of cultural traits and all four preservation bias factors (Conservation: ρ = −0.046, p = 0.647; Continuity: ρ = −0.118, p = 0.234; Number of Occurrences: ρ = −0.028, p = 0.784; Detectability: ρ = −0.097, p = 0.324). These results indicate that preservation biases do not strongly influence the apparent timing of cultural trait emergence in the archaeological record.

Similarly, multiple linear regression analysis (S2 File) demonstrated that preservation bias factors do not significantly predict the first appearance of cultural traits (F(4,98) = 0.812, p = 0.520, R² = 0.032). None of the individual bias factors exhibited significant effects (p > 0.05 for all predictors), further supporting the conclusion that preservation biases do not systematically distort the chronological order of cultural trait emergence.

The random perturbation model demonstrated that the relative ranking of cultural traits is highly stable despite realistic dating uncertainties. The Spearman rank correlation between the original and perturbed rankings was 0.989 (p < 0.001), indicating that while minor shifts occur, the overall chronological sequence remains robust.

To test the stability of our temporal partitions, we systematically shifted the boundaries of our 9 predefined time periods by ±10% and ±20% and reassigned cultural traits accordingly (S2 File). With a + 10% shift, 92.23% of cultural traits remained in their original time period. With a −10% shift, 80.58%. With a + 20% shift, 79.61%; and with a −20%, 61.17%. These results indicate that the vast majority of cultural traits remain consistently classified even when moderate shifts are introduced. While some traits that are near boundary edges do shift between adjacent periods, the overall classification remains stable. To further assess the statistical robustness of these classifications (see Methods), we performed Chi-square tests for independence comparing the original and shifted classifications. For the + 10% shift: Chi-square value: 694.17; p-value: < 2.2^-16^ (highly significant); For the −10% shift: Chi-square value: 570.81; p-value: < 2.2^-16^ (highly significant). For the + 20% shift: Chi-square value: 522.7; p-value: < 2.2^-16^ (highly significant); For the −20% shift: Chi-square value: 448.9; p-value: < 2.2^-16^. These results strongly indicate that the classification of cultural traits into time periods is not random and retains significant structure even when adjusted, confirming that our temporal framework is not an arbitrary division but a robust representation of cultural evolution trends.

### Evolutionary trends in the spatial, temporal and social dimensions of cultural transmission

The relevance taken by different modes of cultural transmission through time (Fig 1) highlights discernible trends in the spatial and temporal dimensions of the practitioner-learner interactions, while presenting a more complex pattern in the social dimension of cultural transmission.

In the spatial dimension (Fig 1a), the distal observation mode (SPAdistal) appears to be a minor transmission strategy before 2My and to play a negligible role thereafter. During this period proximate-disconnected (SPAproDis) and proximate-conscious observation (SPAproCon) dominates the picture. Appearing before 2.8My, the proximate observation with practitioner intentionality (SPAproInt) takes center stage as the primary mode of cultural transmission between about 2My and 400ky while remaining markedly active up to 65ky. A significant shift occurs around 400ky when explanation complemented action (SPAexpAct), already at work after 2.4My, starts playing a crucial role, becoming the key mode of cultural transmission from ca. 300ky to the end of the Paleolithic. With only minor exceptions before 100ky, explanation in absence of action (SPAabsAct) is discerned in material culture after about 75ky, gaining increasing relevance after 50ky.

In the temporal dimension (Fig 1b), the single transfer mode (TEsingle), attested since 3.3My, gradually diminishes in significance after 2My, yet continues to play some role in transmitting certain cultural traits throughout the Paleolithic. The repetitive transfer mode (TErepeti) dominates the learner-practitioner interaction between 3.3My and 300ky, gradually losing relevance between 280ky and 70ky, but remaining in use up to the end of the Paleolithic. The sequential transfer mode (TEsequent) progressively gains importance starting from 2.3My and especially after 200ky. Although sporadically inferred between 600ky and 200ky and more relevant up to about 75ky, the modular transfer mode (TEmodular) becomes the predominant mode of cultural transmission after 75ky. The disconnected mode (TEdisco) is only sporadically observed before approximately 70ky and, although becoming more relevant after this time, does not seem to dominate in any period of the Paleolithic.

In the social dimension (Fig 1c), non-selective modes (SOhoVeOb, SOhyObGe) dominate the picture between 3.3My and 400ky, with selective modes (SOhoVeObSe, SOhyObSe) playing negligible role in this period. After 400ky, non-selective modes remain consistently in use throughout the Paleolithic with the selective modes, and in particular the non-generalized selective one (SOhoVeObSe), gradually gaining relevance from 400ky and especially after 100ky.

A striking asynchronicity is observed in the main transitions within the spatial and the temporal dimensions. Specifically, in the spatial dimension, the transition from proximate observation with practitioner intentionality to explanation complemented action unfolds between 400ky and 300ky. In contrast, within the temporal dimension, the cessation of dominance by repetitive modes takes place at around 200ky. In the ensuing period (200ky-80ky), the sequential mode appears to assume prominence before giving way to the eventual predominance of the modular mode.

### Correlations between the modes of cultural transmission within and across dimensions

Correlations between modes of cultural transmission (Fig 2; S1 Fig reports the correlation values) shed light on the interplay of spatial, temporal, and social dimensions in the transmission of cultural traits. Within the spatial dimension, modes of cultural transmission involving mere observation (SPAdistal, SPAproDis, SPAproCon) exhibit strong correlations, suggesting a commonality in the dynamics of distant and proximal observation. Similarly, modes rooted in gesture molding (SPAgeMo, SPAgeCo) display significant correlations, indicating a similar role in transmitting cultural traits. Explanation complemented action (SPAexpAct) and explanation in absence of action (SPAabsAct) are positively correlated. However, both are anti-correlated with modes involving mere observation, highlighting a dichotomy between instructive, explanation-based modes and observational ones. Within the temporal dimension, simple and repetitive modes (TEsimple, TErepetitive) are positively correlated. Conversely, the most structured modes (TEsequent, TEmodular, TEdisco) exhibit relevant correlations among themselves. These structured modes are anti-correlated with simple and repetitive modes, indicating a dichotomy between hierarchical and straightforward transmission. Within the social dimension, selective modes (SOhoVeObSe, SOhyObSe) are strongly anti-correlated with non-selective modes (SOhoVeOb, SOhyOb), indicating a clear distinction in information flow based on the selection of addressed learners. The two selective modes show correlations with each other.

When exploring interdimensional relationships, spatial modes involving explanation (SPAexpAct and SPAabsAct) are strongly correlated with the most structured temporal modes and the social selective modes. Anti-correlations across dimensions identify complex relationships: spatial modes relying on explanation (SPAexpAct and SPAabsAct) are anti-correlated with simple and repetitive temporal modes (TEsimple, TErepetitive) and non-selective social modes (SOhoVeOb, SOhyObGe). Spatial observation modes (SPAdistal, SPAproDis, SPAproCon) are anti-correlated with structured temporal modes (TEsequent, TEmodular, TEdisco) and social selective modes (SOhoVeObSe, SOhyObSe). Single and repetitive temporal modes (TEsimple, TErepetitive) are anti-correlated with the non-generalized selective social mode (SOhoVeObSe). Structured temporal modes (TEmodular and TEdisco) are anti-correlated with non-selective social modes (SOhoVeOb and SOhyOb).

### Evolution of cultural transmission modes by time periods

Exploring the relevance of distinct modes of cultural transmission in each time period identifies compelling evolutionary trends (Figs 3–5; see also S3 File). In the spatial dimension (Fig 3), between 3.3My and 600ky there is a noticeable decline in the relevance of learning by observation (SPAdistal, SPAproDis, SPAproCon), followed by a near-disappearance of SPAdistal and SPAproDis, with SPAproCon stabilizing at a very low level. Concurrently, there is a gradual raise in the explanation complemented action mode (SPAexpAct). Explanation in absence of action (SPAabsAct) becomes increasingly relevant only from 300ky, never emerging as a primary transmission mode in the Paleolithic. Proximate observation with practitioner intentionality (SPAproInt) experiences a significant surge between 3.3My and 400ky, gradually decreasing thereafter. The impact of gesture molding modes (SPAgeMo and SPAgeCo) raise from 3.3My to 600ky remaining stable afterwards with a noticeable decrease after 200ky especially in SPAgeMo; however, these two modes appear not to play a prominent role overall.

In the temporal dimension (Fig 4), a decline is observed in the single and repetitive information transfer modes (TEsingle and TErepetitive) accompanied by an increase in other cultural transmission modes (TEdisco, TEmodular and TEsequent). TEsequent steadily increases between 3.3My and 300ky, whereas the modular mode surges remarkably after 300ky. The disconnected mode (TEdisco) appears to play a minor role only after 200ky.

A contrasting trend is observed in the social dimension (Fig 5) between non-selective (SOhoVeOb and SOhyObGe) and selective modes (SOhoVeObSe and SOhyObSe). The former slightly decrease in time overall, though following an alternating and peaked pattern. The selective modes tend to increase appreciably over time, also showing an alternating pattern. Of note that selective and non-selective modes reach a near equilibrium after 100ky and more markedly in the last 50ky. The reciprocal social modes do not appear to play a role in the Paleolithic.

### Discriminant analysis of cultural traits

The discriminant analysis (Fig 6), encompassing all cultural traits categorized by period and all modes of cultural transmission (excluding the social reciprocal modes: SOhyObReGe, SOhyObReSe) successfully accounted for 77,21% of the variation. The first canonical axis, contributing significantly with 60.97%, organizes cultural traits from the earliest to the more recent periods. The convex hulls for each period demonstrate variable overlap with adjacent periods, and very little overlap between more distant time frames. The modes of cultural transmission that exhibit the highest loadings, contributing significantly to the differentiation of cultural traits from the earliest phases, include SPAproCon, TErepeti, TEsingle, SOhyObGe, and SOhoVeOb. Conversely, modes identifying more recent cultural traits comprise SPAexpAct, SPAabsAct, TEmodular, TEdisco, TEsequent, SOhoVeObSe, and SOhyObSe. The second canonical axis is particularly influential in distinguishing traits that emerged between 3.3–2.8My from those emerging 2.8–2My and the latter form those raising at 2My-600ky. The modes of cultural transmission discriminating cultural traits along the second axis include SPAproInt, SPAdistal, SPAproDis, SPAgeMo and SPAgeCo.

Looking at the distribution of variables (i.e., the considered cultural transmission modes), axis 1 captures the increasing amount of communication and overt explanation between practitioner and learner needed for effective transmission of a cultural trait, whereas axis 2 captures the level of concrete interaction between practitioner and learner implicated in successful cultural transmission. The confusion matrix (see S4 Table) demonstrates that, based on the scores attributed to the various modes of cultural transmission for each cultural trait, the discriminant analysis would attribute a trait to the time period in which it actually emerged in 64,08% of cases. This level of confusion is likely due to the imbalanced number of cultural traits belonging to different time periods and, more importantly, to the possibility that a cultural trait was transmitted in a more recent time period by resorting to modes of cultural transmission emerged earlier.

The centroids of the convex hulls (Fig 7) reveal a clear trend, grouping the nine considered time-periods into three distinguishable subsets. The most ancient periods (ranging from 3.3My to 600ky) display a low level of communication and overt explanation (axis 1), along with a scattered level of concrete interactions between practitioner and learner (axis 2), which increases over time. The three time-periods going from 600ky to 200ky present slightly higher communication requirements compared to the previous periods, and share a similar level of concrete interaction. The most recent periods (after 200ky) show markedly increased requirements of communication and overt explanation, which continue to increase over time across these periods, along with a common level of concrete interaction, slightly lower than the previous three periods.

### Seriation

The pattern emerging from the seriation (Fig 8) associates the most ancient cultural traits with the simplest transmission modes (SPAproDis, TEsingle, SPAproCon, SOhoVeOb, TErepetitive, SPAproInt and SOhyObGe), and the most recent traits with the most complex transmission modes (SPAexpAct, SOhoVeObSe, TEmodular, SOhyObSe). Within this general pattern, three groups of cultural traits can be discerned. The first group is associated with 7 modes of cultural transmission (SPAproDis, TEsingle, SPAproCon, SOhoVeOb, TErepeti, SPAproInt, SOhyObGe), and includes traits emerging earlier than 300ky, with three exceptions (two emerging exactly at 300ky and only one after 200ky). At the opposite end of the seriation, a group of cultural traits is characterized by 8 modes of cultural transmission (SPAgeCo, TEsequent, TEdisco, SPAabsAct, SPAexpAct, SOhoVeObSe, TEmodular, SOhyObSe). This group only includes traits more recent than 100ky, with two exceptions (of traits emerging at 130–140ky). In between the two groups lies a third group in which most of the transmission modes present in the other two groups are observed, with the exception of SPAproDis and TEsingle (the latter being represented in just 1 over 45 traits). In this group, only 4 traits older than 400ky are present. Interestingly, the transmission modes associated with the first (top) group of cultural traits in the seriation turn out to be highly correlated among each other (Fig 2 and S1 Fig). The same is observed for the transmission modes associated with the last (bottom) group of traits.

## Discussion

Our results provide an empirically based scenario for the evolution of the strategies employed by members of our lineage to transmit cultural traits in the last 3.3My.

The statistical analyses reported above confirm that the reconstructed chronological framework for cultural transmission in the Paleolithic is robust. The introduction of randomized dating perturbations (with ±10% and ±20% shifts applied to different traits) demonstrates that minor rank order shifts occur, but the overall evolutionary sequence is preserved. The stability of cultural trait rankings even under realistic dating uncertainty suggests that broad evolutionary patterns are not artifacts of dating inaccuracies. This finding reinforces the reliability of our model, suggesting that the inferred sequence of cultural innovations is not an artifact of dating uncertainties. Furthermore, it supports our contention that even under conditions of temporal uncertainty, the general pattern of increasing social and cognitive complexity in the hominin lineage is well preserved.

Our statistical analyses also indicate that preservation biases do not exert a significant effect on the chronological pattern of cultural evolution documented in our dataset. Neither correlation analysis nor regression modeling revealed meaningful relationships between preservation bias factors and the first appearance age of cultural traits. This suggests that while individual cases of preservation bias may exist, their impact is not systematic enough to alter the broad temporal trends observed in our dataset.

These findings align with prior discussions on the robustness of cultural trait chronologies in the archaeological record [19–21]. While some cultural traits—especially those reliant on perishable materials—may have appeared earlier than their first documented occurrence, our dataset incorporates traits that are both highly perishable and well-preserved, reducing the likelihood of a systematic preservation bias affecting the overall pattern. Furthermore, the high continuity of many traits and their recurrence across multiple contexts support the validity of our reconstructed sequence of cultural evolution.

Thus, while we acknowledge the theoretical possibility of preservation biases influencing individual cases, our results provide empirical support that such biases do not significantly distort the broad temporal trends of cultural transmission and innovation in human prehistory. This strengthens confidence in the chronological framework of our study and its implications for understanding the evolution of cultural learning mechanisms over time.

A critical concern in studies of cultural evolution is whether temporal partitions are robust to dating uncertainties. The temporal boundaries (time periods) adopted in this study integrate considerations of data density, archaeological milestones, and transparency. While aggregated trends are analyzed using these intervals, raw trait-specific results are also presented, allowing readers to independently assess the variability and patterns of individual traits. This dual approach minimizes interpretive biases while exploring evolutionary trends. To quantitatively assess this issue, we tested the stability of our predefined time periods by shifting their boundaries by ±10% and ±20% and reassigning cultural traits accordingly. Our results show that most traits (92.23% with a + 10% shift and 80.58% with a −10% shift) remained within their original classification, indicating that our framework is largely insensitive to moderate boundary variations. These results align with those of Chi-square tests. The highly significant results (p < 2.2 ⁻ ^16^ for both ±10% and ±20% shifts) demonstrate that the classification of cultural traits remains highly structured even when moderate changes are introduced. This confirms that the observed patterns of cultural transmission are not artifacts of arbitrary time-period selection but reflect a meaningful evolutionary structure.

These findings reinforce the validity and robustness of our time periods and confirm that they provide a sound framework for investigating the evolution of cultural transmission over the past 3.3 million years. Overall, our findings broadly align with those of Paige and Perreault [17], who demonstrated that cumulative cultural complexity increased over the Paleolithic, particularly during the Middle Pleistocene. Their analysis shows a pattern similar to ours, emphasizing the gradual accumulation of cultural traits and the acceleration of innovation towards the Late Pleistocene. This concurrence supports the view that cumulative culture is a long-term process shaped by increasingly complex interactions between practitioners and learners. However, while Paige and Perreault focus on the macro-patterns of stone tool complexity, our study goes beyond this by analyzing a broader range of cultural traits and of the transmission strategies themselves, addressing the mechanisms through which cultural traits were perpetuated and refined. Our research extends beyond the well-established conclusion that culture accumulates over time and accelerates towards the Late Pleistocene. By disentangling the spatial, temporal, and social dimensions of transmission, we reveal how these modes evolved in response to the increasing complexity of cultural traits. For example, we identify timelines for shifts from distal observation to explanation-based modes (e.g., SPAexpAct, SPAgeCo), the emergence of modular and disconnected temporal strategies, and the rise of selective social transmission. These findings highlight how cultural innovations shaped—and were shaped by—changes in transmission strategies. Furthermore, the temporal framework we provide, including the delays between shifts in spatial and temporal transmission modes, offers insights into the co-evolution of cultural complexity and communication.

The absence of distal observation as a dominant mode even at 3.3 million years ago suggests that early cultural traits required engaged transmission strategies. This indicates that already 3.3My cultural traits associated with our ancestors had reached a degree of complexity requiring more than just learning through observation from a distance, a transmission strategy widespread in apes and other primates [95–97].

In the considered period (3.3My-6ky), no abrupt change is observed at any time in the way knowledge was transmitted. We observe instead a pattern of changing preferences in cultural transmission strategies, with more complex modes gradually taking precedence across the spatial, temporal, and social dimensions of practitioner-learner interaction. This gradual pattern appears incompatible with the view that a single biological change could have been at the origin of “modern human cognition”. Our findings support two overarching trends: first, a shift from high spatial proximity and simple observation towards more intentional and explanatory interactions; second, a transition from repetitive to structured and modular temporal modes. These changes coincide with the increasing complexity of cultural traits and social organization, particularly between 600,000 and 100,000 years ago.

This period represents, unlike the previous and later periods, a phase in which almost all modes of transmission are employed as essential means to perpetuate cultural traits (Figs 6–8). This phase reflects a process in which the emergence of an increasing number of cultural innovations was accompanied by the exploration of novel strategies to transmit them. By 100,000 years ago, cultural transmission strategies consolidated around modes involving overt explanation, structured temporal sequences, and selective social pathways. Interestingly, we observe (Fig 1) a delay between the transition from observation-based to explanation-based spatial modes (occurring at ca. 400ky) and the transition from simple to structured temporal mode (observed at ca. at 200ky). This might be explained considering that structured temporal transmission requires the previous development of sustained and effective communication between practitioner and learner.

In the social dimension (Fig 1c), our results show a raise in the social selective modes (those in which information is transferred from specialized practitioners to selected learners) starting at about 400ky and becoming widespread after 100ky, when selective and non-selective modes reach an equilibrium (Fig 5). This suggests that at that time culture was complex enough to require division of labor. In such situation, social learning strategies (SLSs) acquire a specific relevance as learners must be able to appropriately select what to learn and from whom [33,35]. This also has implications for the construction of human cultural niches characterized by distinct but interacting sectors of expertise and knowledge.

Perpetuation of innovations stemming from Type II CCE [13], characterized by the combination of natural phenomena of different kinds to obtain novel cultural traits, generally requires learning discrete modules of information in a stepwise manner. The growing prominence of modular and disconnected temporal modes from 200,000–75,000 years ago aligns with the rise of Type II cumulative cultural evolution, involving the integration of diverse natural phenomena into novel traits [13].

In the spatial dimension (Fig 1a), our results unveil that explanation complemented action increases in relevance after ca. 500ky and becomes the main transmission mode at ca. 200ky. This might be related to the increasing opacity of the physical properties and procedures involved in the production and use of artefacts. Hence, devising transmission strategies able to overcome this problem became a key challenge. This might have exerted a pressure for experts to develop effective teaching strategies aimed at facilitating pupils’ social learning [46]. The increasing reliance on explanation-based spatial modes, sequential temporal modes, and selective social pathways suggests growing communication requirements between 500ky and 250ky, perhaps associating gesture and verbal expressions.

Between 200ky and 75ky, the dominance of explanation-based and modular strategies points to the emergence of modern language. This is consistent with the pattern identified by the discriminant analysis (Figs 6 and 7), with a marked increase in the communication requirement after 200ky, which keeps increasing thereafter. It also aligns with the “technological hypothesis” for language evolution [98,99], which posits that the need to transmit tool-making skills drove linguistic advancements, but also calls for its expansion in two directions. First, our results suggest that a significant pressure on effective communication was exerted by more symbolic and socially relevant cultural traits such as ornaments, pigments, expressive and notational artefacts, complex mortuary practices and exchange of rare or exotic goods. Hence, the technological hypothesis should be extended to a truly pedagogical hypothesis for language evolution [38] according to which language evolution was prompted by cultural traits beyond tool-making. Second, our results suggest that the emergence of modern articulate language might not be the result of an abrupt change in brain and cognition, but should be regarded as a gradual process driven by behavioral and cultural factors [10,84,100–103]. According to the results presented here, these factors concern the complexification of culture and the consequent need for finer cultural transmission strategies (Fig 8).

Our findings contribute to the ongoing debate on the emergence and evolution of cultural transmission strategies in the human lineage by providing an empirically grounded scenario for their development. The results highlight a long-term co-evolutionary process between the emergence of cultural traits and the increasing complexity of transmission mechanisms. These findings have implications for our understanding of the origins of behavioral modernity, particularly in relation to the long-standing discussion regarding a gradualist versus punctuated emergence of symbolic behaviors and technological complexity.

Contrasting our results to all models proposed in the past to account for the emergence of cultural modernity is out of the scope of the present work and could certainly be pursued in the future. However, to situate our findings within the broader discourse, we briefly outline key perspectives on the emergence of modern human behavior. Traditionally, models of modern human behavior have oscillated between revolutionary and gradualist interpretations. McBrearty and Brooks [104] challenged the notion of a sudden ‘behavioral revolution’ coinciding with the European Upper Paleolithic, proposing instead that key components of behavioral modernity emerged gradually in Africa over a longer time span. This perspective has been further refined by Scerri and Will [105], who argue that behavioral complexity in *Homo sapiens* was not a singular event but a distributed, mosaic process, shaped by demographic and ecological variability across African populations. Our findings align with these views by demonstrating that cultural transmission strategies evolved incrementally and in response to the increasing complexity of cultural traits, rather than appearing abruptly as a singular package.

At the same time, the punctuated “multi-species” model proposed by other authors [106–108] emphasizes that comparable cultural innovations are found among populations attributed in the past to different species and that not always innovations spread homogeneously but emerged in discontinuous bursts, often associated with ecological or demographic shifts. This perspective highlights that cultural transmission strategies must be understood within a framework that considers environmental constraints, population structures, and mechanisms of intergroup learning. Our study provides empirical support for this view by showing that while transmission strategies evolved progressively, there were periods of acceleration, particularly around 300–200 ka and 100–50 ka, when significant shifts in cultural complexity and transmission mechanisms occurred.

The distinction between different cultural transmission modes also has implications for understanding the role of niche construction in hominin evolution. Conard [109] proposed that the cultural evolution of Middle and Late Pleistocene hominins was shaped by their ability to modify ecological and social environments through cumulative cultural practices. Our results provide further evidence that as transmission strategies evolved, they facilitated greater degrees of cultural niche construction. This supports the argument that hominin cultures did not merely adapt passively to environmental pressures but actively reshaped their selective landscapes through innovations in material culture and social learning.

Furthermore, our results complement the theoretical framework proposed by Colagè and d’Errico [10], which highlights culture as a driving force in cognitive evolution. As our study suggests, changes in transmission strategies influenced not only the spread of cultural traits but also the cognitive and social capacities required for learning, teaching, and innovation. The increasing role of structured learning environments, as evidenced by the rise of modular and sequential transmission modes, supports the notion that cultural scaffolding played a crucial role in shaping the cognitive architecture of *Homo sapiens*.

Taken together, these perspectives emphasize the need to move beyond simplistic dichotomies of ‘modern’ versus ‘non-modern’ behavior. Instead, cultural evolution should be viewed as a dynamic process influenced by ecological variability, demographic structures, and the continuous feedback between innovation and transmission. The interplay between cultural trait complexity and evolving transmission strategies provides a new lens to understanding the emergence of human uniqueness. Our findings underscore the importance of refining models of cultural transmission by integrating archaeological, ethnographic, and experimental approaches to reconstruct past learning processes with greater accuracy.

Two avenues for future research stand out. First, comparisons with trends in encephalization rates could elucidate potential co-evolutionary dynamics between brain expansion and the increasing complexity of cultural transmission strategies. Understanding these links could clarify how biological and cultural factors interacted to shape human evolution. Investigating whether the emergence of more structured and cumulative forms of cultural transmission correlates with specific milestones in hominin brain evolution—such as increases in frontal lobe size or connectivity—could offer valuable insights into the cognitive demands of evolving cultural complexity. Additionally, examining fossil evidence for changes in brain organization alongside archaeological indicators of teaching and learning could help establish clearer causal links between brain expansion and cultural transmission.

Second, exploring correlations with climatic variability offers another promising perspective. Climate shifts have been proposed as drivers of innovation and social reorganization, and examining their relationship with transmission strategies could provide deeper insights into the adaptive contexts of cumulative culture. For instance, the increased frequency of climatic fluctuations during the Middle and Late Pleistocene may have played a role in selecting for more flexible and efficient learning mechanisms. Comparing periods of cultural acceleration with known climatic transitions could help disentangle whether environmental instability acted as a catalyst for more effective cultural transmission strategies. By integrating these biological and environmental perspectives, future research could provide a more comprehensive understanding of how human cultural complexity emerged and stabilized over evolutionary time.

## Supporting information

S1 Fig Correlation values among variables. Correlation values among the 19 modes of cultural transmission considered in this study (see *Methods* for the definitions of these variables). These values are the same with which Main Text’s Fig 2 is produced.
(TIF)

S1 Table Cultural traits and descriptions. This Table presents the 103 cultural traits considered in this study, divided into categories, with their abbreviation, the time of first consolidated appearance and the appropriate time period for statistical analysis as in Main Text’s Table 1. A definition of each cultural trait and key, selected references for the date of first consolidate appearance in the archaeological record are also provided. The references for the date of first consolidate appearance of each trait reported in this table (column F) are listed below the table.
(XLSX)

S2 Table Score changes after discussion with experts. The Table reports the changes we made on the database scores (see S1 File) after discussion with experts. See Main Text’s *Methods* for the overall score assignment procedure.
(XLSX)

S3 Table Scores of preservation and detectability factors. The Table reports the scores given to each of the four preservation biases (Conservation in the archaeological record, Continuity in the archaeological record, Number of occurrences, Detectability) for each cultural trait considered in this study. See Main Text’s *Methods* for definition of the four biases.
(XLSX)

S4 Table Confusion matrix for discriminant analysis. This Table reports the number of cultural traits that the discriminant analysis (see Main Text’s Fig 6) would have attributed to a different time period than the one we assigned. The greatest discrepancies (highlighted in light and dark pink) are found in the most recent periods.
(XLSX)

S1 File Formatted database. This Excel spreadsheet contains the entire database for this study, including the scores assigned to each cultural transmission mode (variables; columns E-W) for each cultural trait (individuals; lines 2–104). The scores reported are the final ones (after the three steps for score assignment – see Main Text’s *Methods*) used in all the analyses. For each trait, the abbreviation (column B), the age of first consolidate appearance (column C), and the appropriate time period (column D) are provided. See Main Text’s *Methods* for the definition of the variables; Tables 1 and S1 provide further details on the considered cultural traits.
(XLSX)

S2 File Assessment of preservation biases and dating robustness. This file reports the statistical analysis on the scores assigned (see S3 Table) for each considered cultural trait to the four preservation biases (Conservation in the archaeological record, Continuity in the archaeological record, Number of occurrences, Detectability), as defined in the Main Text’s *Methods*. The R scripts used, the results obtained and related plots are all included in this file. This file also reports the robustness tests performed both on the date of first appearance for each cultural trait, and on the time periods used for this study (see Tables 1, S1, and Main Text’s *Methods*). The R scripts used, the results obtained and related plots are all included in this file.
(DOCX)

S3 File Variables-within-period mean values and standard errors. This Excel spreadsheet contains the data and calculations for the mean values and standard errors of the variables used to produce Figs 3–5 in the Main Text. The cultural traits in this sheet are grouped according to their appropriate time period. For each trait, the abbreviation (column B), the age of first consolidate appearance (column C), and the appropriate time period (column D) are provided. See Main Text’s *Methods* for the definition of the variables; Tables 1 and S1 provide further details on the considered cultural traits.
(XLSX)

S4 File Presence/absence matrix for seriation. This Excel spreadsheet contains the database scores used for the seriation, where all values of 4 have been transformed into 1, and all values of 1, 2 and 3 have been transformed into 0. Values of 0 remained unchanged (see Main Text’s Fig 8). The cultural traits in this sheet are grouped according to their appropriate time period. For each trait, the abbreviation (column B), the age of first consolidated appearance (column C), and the appropriate time period (column D) are provided. See the Main Text’s *Methods* for the definition of the variables; Tables 1 and S1 provide further details on the considered cultural traits.
(XLSX)
