# Supplementary figures and images for: An empirically-based scenario for the evolution of cultural transmission in the human lineage during the last 3.3 million years
Source: PLoS One. 2025 Jun 4;20(6):e0325059. doi: 10.1371/journal.pone.0325059 (PMC12136325; doi:10.1371/journal.pone.0325059)

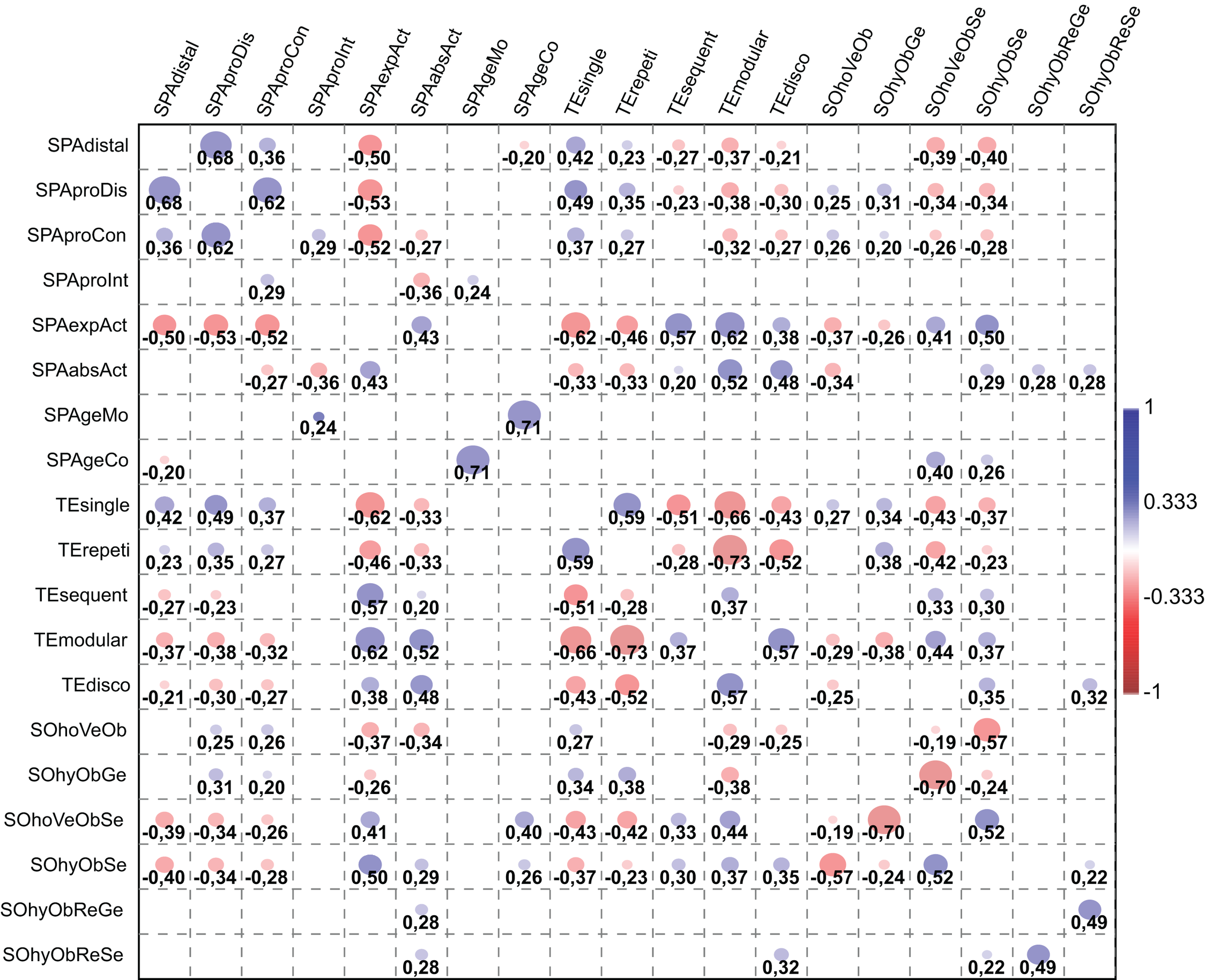

Supplement: S1 Fig — Correlation values among the 19 modes of cultural transmission considered in this study (see Methods for the definitions of these variables). These values are the same with which Main Text’s Fig 2 is produced. (TIF) [file pone.0325059.s001.tif]
