# Supplementary material for: An empirically-based scenario for the evolution of cultural transmission in the human lineage during the last 3.3 million years
Source: PLoS One. 2025 Jun 4;20(6):e0325059. doi: 10.1371/journal.pone.0325059 (PMC12136325; doi:10.1371/journal.pone.0325059)
Supplement: S2 File — This file reports the statistical analysis on the scores assigned (see S3 Table) for each considered cultural trait to the four preservation biases (Conservation in the archaeological record, Continuity in the archaeological record, Number of occurrences, Detectability), as defined in the Main Text’s Methods. The R scripts used, the results obtained and related plots are all included in this file. This file also reports the robustness tests performed both on the date of first appearance for each cultural trait, and on the time periods used for this study (see Tables 1, S1, and Main Text’s Methods). The R scripts used, the results obtained and related plots are all included in this file. (DOCX) [file pone.0325059.s007.docx]

Assessment of preservation biases and dating robustness

Table of contents

[1. Loading and preparing data 1](#_Toc194789257)

[2. Preservation biases 2](#_Toc194789258)

[3. Robustness of the order of appearance of the cultural traits 7](#_Toc194789259)

[4. Robustness of the period’s attribution of the cultural traits 8](#_Toc194789260)

# Loading and preparing data

In this section, we load the necessary libraries and prepare the dataset for analysis. The data is sourced from an Excel file (see S3 Table) and includes information on various cultural traits. Column names are standardized for clarity, and relevant fields are converted to numeric format for further analysis. We then plot a histogram showing the distribution of the first appearance of cultural traits over time.

# Load necessary libraries
library(readxl)
library(ggplot2)
library(corrplot)
library(dplyr)
library(Hmisc)
library(car)
library(gmodels)

# Load and prepare dataset
dataset <- read_excel("~/Recherche/Francesco dErrico/script R test/SupplementaryTable1_CulturalTraitsDescriptionsPlusScoresAfterIvanFinal.xlsx",sheet = "Cultural Traits Table")

# Rename columns for clarity
colnames(dataset) <- c("Category", "Cultural_Trait", "Label", "First_Appearance_Age", "Time_Period",
 "References", "Description", "Conservation", "Continuity", "Number_of_Occurrences", "Detectability")

# Convert necessary columns to numeric
dataset$First_Appearance_Age <- as.numeric(dataset$First_Appearance_Age)
dataset$Conservation <- as.numeric(dataset$Conservation)
dataset$Continuity <- as.numeric(dataset$Continuity)
dataset$Number_of_Occurrences <- as.numeric(dataset$Number_of_Occurrences)
dataset$Detectability <- as.numeric(dataset$Detectability)

# Plot histogram of cultural traits' first appearance
ggplot(dataset, aes(x = First_Appearance_Age)) +
 geom_histogram(bins = length(levels(as.factor(dataset$First_Appearance_Age))), fill = "blue", color = "black", alpha = 0.7) +
 scale_x_reverse() +
 labs(title = "Distribution of Cultural Traits' First Appearance",
 x = "Age of First Appearance (years)", y = "Frequency")


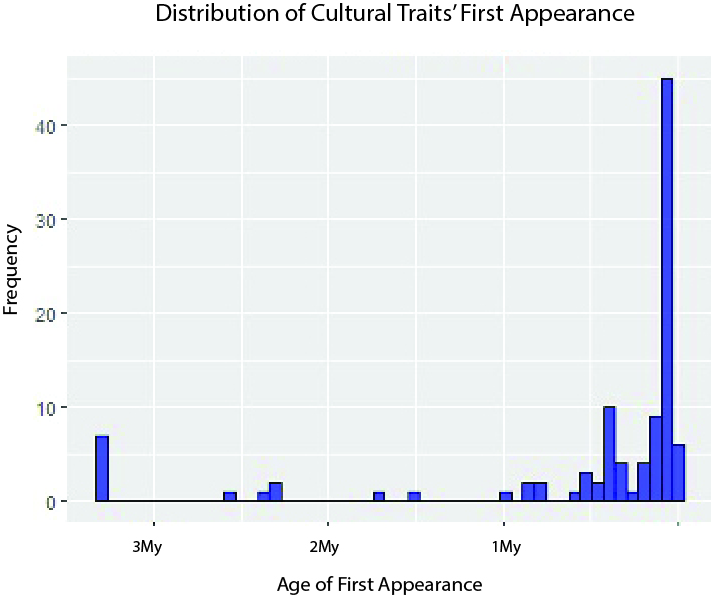


# 2. Preservation biases

In this analysis, we test whether any of four potential preservation biases—*conservation*, *continuity*, *number of occurrences*, and *detectability* (see Main Text’s *Methods* for definitions of these biases)—are correlated with the estimated age of first appearance for each cultural trait. Correlation coefficients are calculated among these variables and displayed in a matrix. The results show very weak negative correlations between first appearance age and the four bias indicators, none of which are statistically significant (p > 0.1). However, strong correlations exist between the bias indicators themselves, particularly between continuity and number of occurrences (r = 0.81, p < 0.0001). A multiple linear regression model was then used to predict the first appearance age based on the four preservation variables. The model explained only a small portion of the variance (Adjusted R² = -0.0074), and none of the predictors were statistically significant. Scatter plots with regression lines are provided to visualize the relationship between each preservation bias variable and the age of first appearance.

# 2. Preservation Bias Effects on Temporal Order
cor_results <- rcorr(as.matrix(dataset[, c("First_Appearance_Age", "Conservation", "Continuity", "Number_of_Occurrences", "Detectability")]))
print(cor_results)

First_Appearance_Age Conservation Continuity
First_Appearance_Age 1.00 -0.05 -0.12
Conservation -0.05 1.00 0.56
Continuity -0.12 0.56 1.00
Number_of_Occurrences -0.03 0.64 0.81
Detectability -0.10 0.47 0.47
 Number_of_Occurrences Detectability
First_Appearance_Age -0.03 -0.10
Conservation 0.64 0.47
Continuity 0.81 0.47
Number_of_Occurrences 1.00 0.46
Detectability 0.46 1.00

n= 103


P
 First_Appearance_Age Conservation Continuity
First_Appearance_Age 0.6460 0.2343
Conservation 0.6460 0.0000
Continuity 0.2343 0.0000
Number_of_Occurrences 0.7773 0.0000 0.0000
Detectability 0.3290 0.0000 0.0000
 Number_of_Occurrences Detectability
First_Appearance_Age 0.7773 0.3290
Conservation 0.0000 0.0000
Continuity 0.0000 0.0000
Number_of_Occurrences 0.0000
Detectability 0.0000

# Visualize correlation matrix
cor_results_forplot <- cor(dataset[, c("First_Appearance_Age", "Conservation", "Continuity", "Number_of_Occurrences", "Detectability")])
corrplot(cor_results_forplot, method = "number", type = "upper")


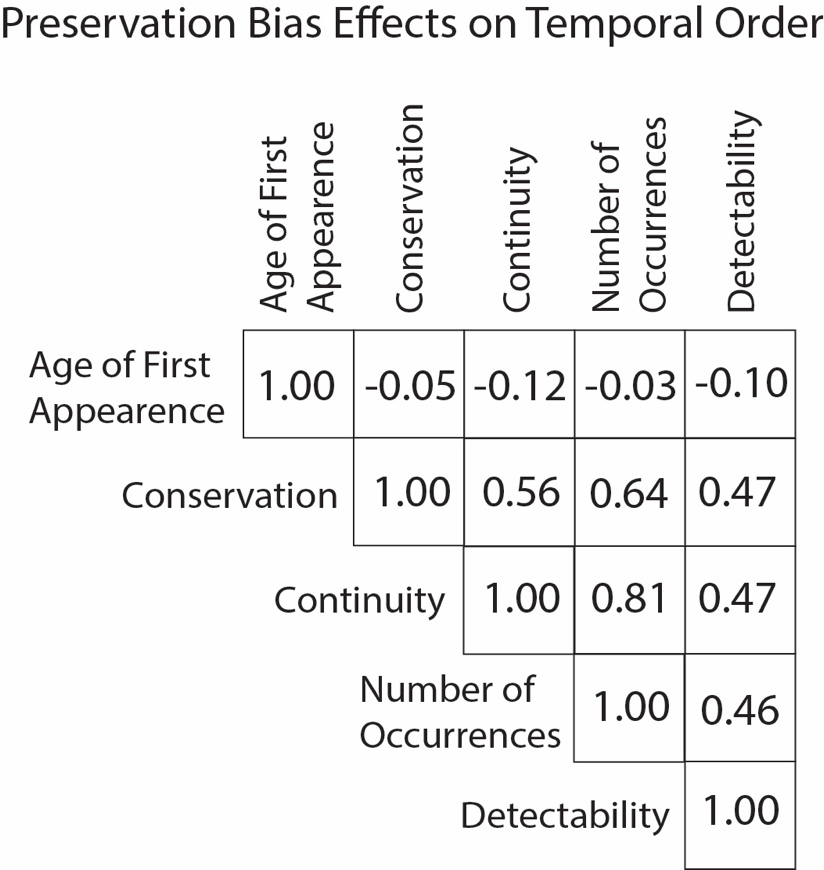


# Multiple Linear Regression for Preservation Bias Prediction
lm_model <- lm(First_Appearance_Age ~ Conservation + Continuity + Number_of_Occurrences + Detectability, data=dataset)
summary(lm_model)

Call:
lm(formula = First_Appearance_Age ~ Conservation + Continuity +
 Number_of_Occurrences + Detectability, data = dataset)

Residuals:
 Min 1Q Median 3Q Max
-931113 -489386 -327346 -160 2973687

Coefficients:
 Estimate Std. Error t value Pr(>|t|)
(Intercept) 645438 170157 3.793 0.000257 ***
Conservation -7343 109327 -0.067 0.946590
Continuity -265750 177348 -1.498 0.137225
Number_of_Occurrences 214484 178427 1.202 0.232226
Detectability -64855 103025 -0.630 0.530479
---
Signif. codes: 0 '***' 0.001 '**' 0.01 '*' 0.05 '.' 0.1 ' ' 1

Residual standard error: 914200 on 98 degrees of freedom
Multiple R-squared: 0.03208, Adjusted R-squared: -0.007422
F-statistic: 0.8121 on 4 and 98 DF, p-value: 0.5204

# Generate scatter plots for visualization
plot1 <- ggplot(dataset, aes(x = Conservation, y = First_Appearance_Age)) +
 geom_point() +
 geom_smooth(method = "lm", col = "blue") +
 ggtitle("Conservation vs. First Appearance Age")

plot2 <- ggplot(dataset, aes(x = Continuity, y = First_Appearance_Age)) +
 geom_point() +
 geom_smooth(method = "lm", col = "blue") +
 ggtitle("Continuity vs. First Appearance Age")

plot3 <- ggplot(dataset, aes(x = Number_of_Occurrences, y = First_Appearance_Age)) +
 geom_point() +
 geom_smooth(method = "lm", col = "blue") +
 ggtitle("Number of Occurrences vs. First Appearance Age")

plot4 <- ggplot(dataset, aes(x = Detectability, y = First_Appearance_Age)) +
 geom_point() +
 geom_smooth(method = "lm", col = "blue") +
 ggtitle("Detectability vs. First Appearance Age")

# Print plots
print(plot1)


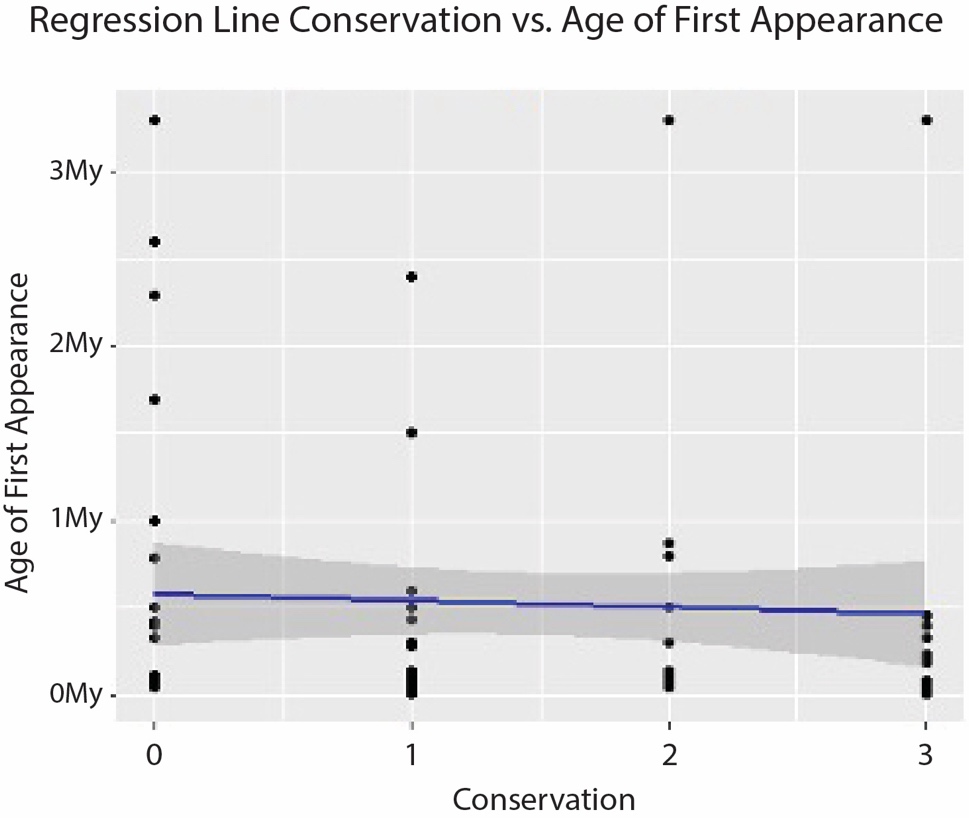


print(plot2)


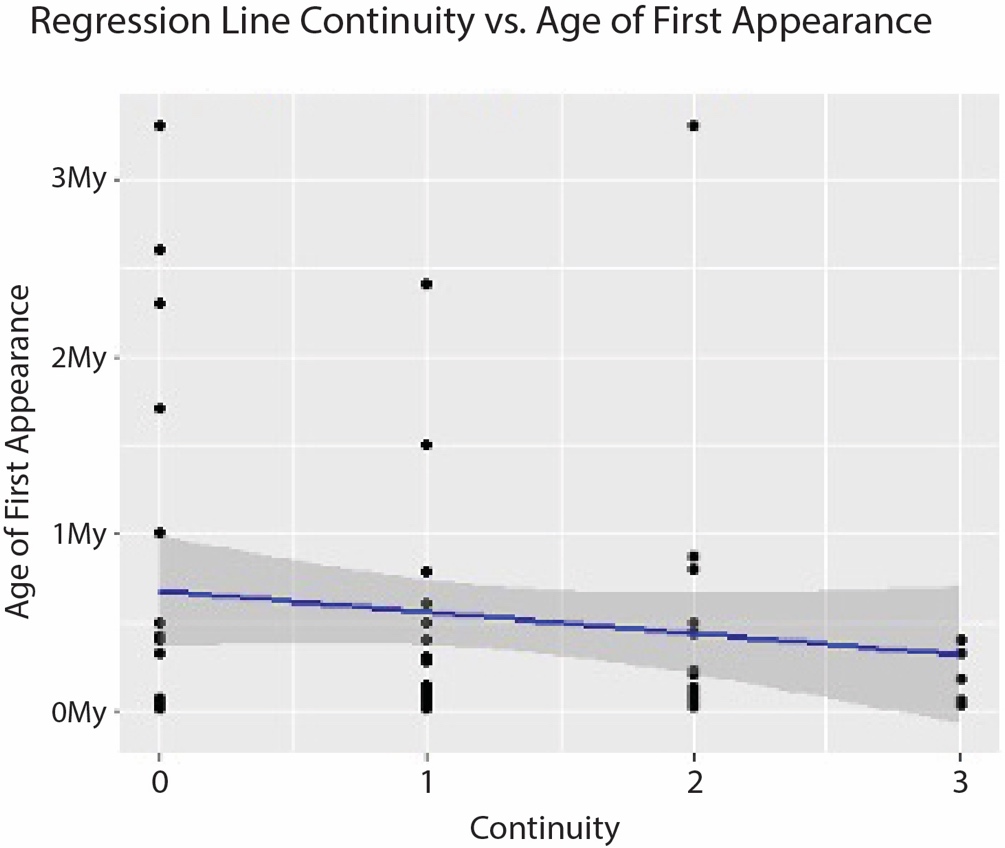


print(plot3)


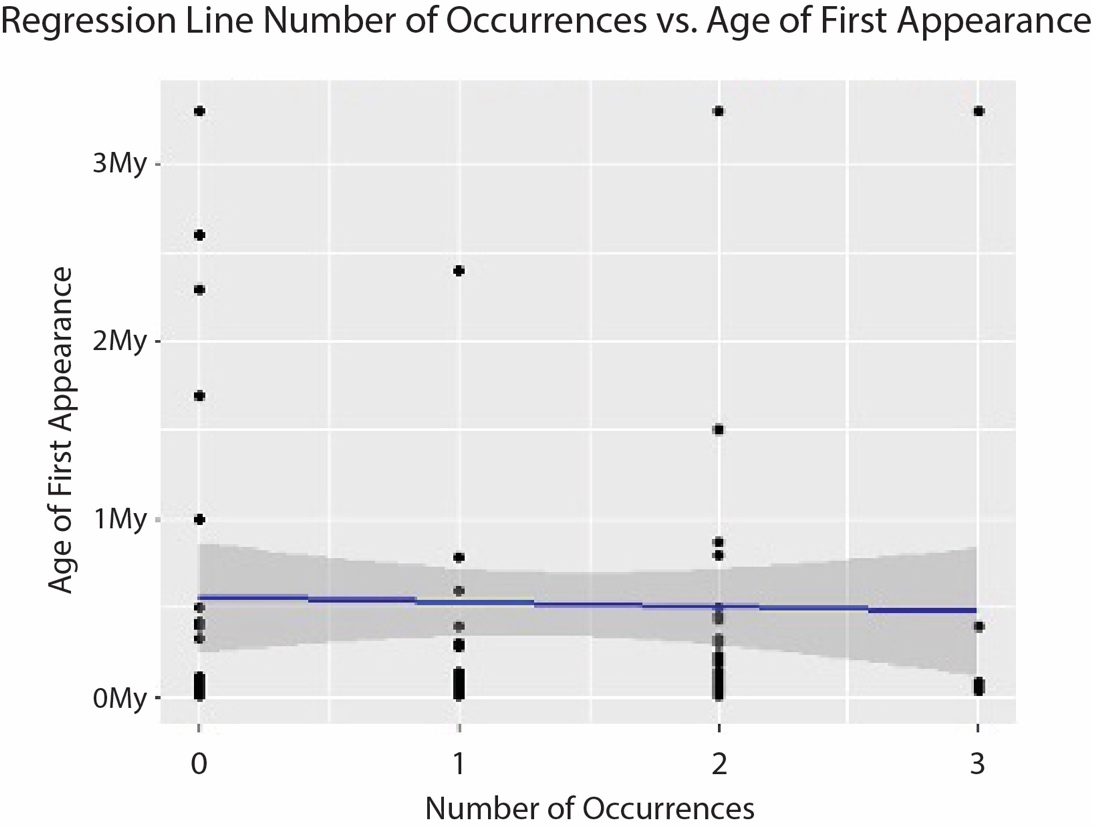


print(plot4)


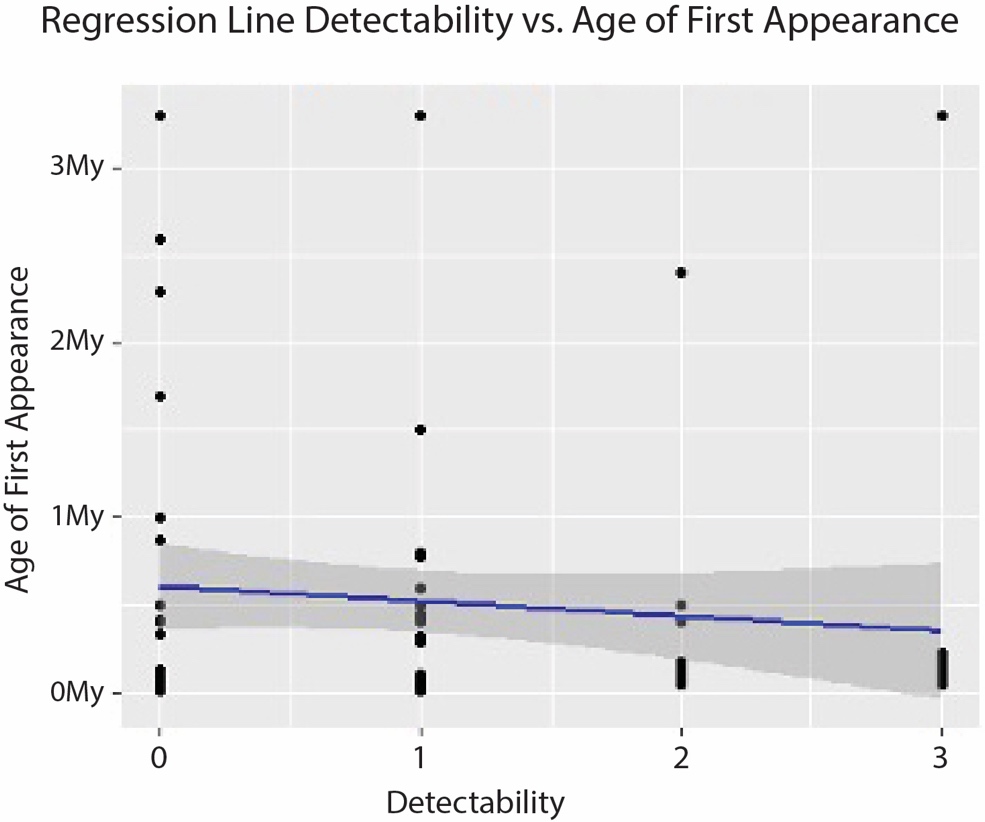


# 3. Robustness of the order of appearance of the cultural traits

This section assesses the robustness of the chronological order of cultural trait emergence by introducing random perturbations to the dates of first appearance. In each of 1,000 simulations: One-third of the traits have their dates modified by ±10%; Another third are adjusted by ±20%; The remaining third remain unaltered. Trait assignment to each perturbation group is randomized in every iteration. For each simulation, the Spearman rank correlation between the original and perturbed datasets is computed. The average correlation across all iterations is **0.989**, indicating that the chronological order of cultural traits is highly robust to such perturbations.

# 2. Random Perturbation Model for Chronological Framework Robustness
coef_cor = matrix(0, nrow = 1000, ncol = 1)
for (seed in 1:1000) {
set.seed(seed) # For reproducibility
perturbed_data <- dataset
num_traits <- nrow(dataset)

# Randomly select 33% of traits for ±10% perturbation
traits_10 <- sample(1:num_traits, size = round(0.33 * num_traits), replace = FALSE)
perturbed_data$First_Appearance_Age[traits_10] <- perturbed_data$First_Appearance_Age[traits_10] * sample(c(0.9, 1.1), length(traits_10), replace = TRUE)

# Randomly select 33% of traits for ±20% perturbation
remaining_traits <- setdiff(1:num_traits, traits_10)
traits_20 <- sample(remaining_traits, size = round(0.33 * num_traits), replace = FALSE)
perturbed_data$First_Appearance_Age[traits_20] <- perturbed_data$First_Appearance_Age[traits_20] * sample(c(0.8, 1.2), length(traits_20), replace = TRUE)

# Compute Spearman Rank Correlation between original and perturbed rankings
correlation_perturbation <- cor(dataset$First_Appearance_Age, perturbed_data$First_Appearance_Age, method="spearman")
coef_cor[seed,1] = correlation_perturbation
#print(correlation_perturbation)
}

print(mean(coef_cor))

[1] 0.989241

# 4. Robustness of the period’s attribution of the cultural traits

This section evaluates whether small changes in the boundaries of defined chronological periods significantly affect the period assignment of cultural traits. We define nine original time periods and test four modified versions by increasing or decreasing their boundaries by 10% and 20%. Due to potential overlap, upper and lower boundaries are adjusted separately. The percentage of agreement between the original and modified period attributions is calculated as follows:

- **+10% shift**: 92.23% agreement
- **-10% shift**: 80.58% agreement
- **+20% shift**: 79.61% agreement
- **-20% shift**: 61.17% agreement

These results indicate that minor changes in period boundaries have a measurable impact on trait attribution, especially when boundaries are lowered. Chi-square tests on contingency tables confirm that the original and perturbed classifications are not statistically independent (p < 2.2e-16 in all cases). Bar plots are provided to visualize the distribution of first appearance periods under each condition. It is noteworthy that **lowering upper boundaries** has a more substantial effect on attribution than lowering lower boundaries. This is likely because many cultural traits have estimated first appearance dates that are close to the upper limits of the original periods.

# 3. Sensitivity Analysis for Time Period Robustness

# Define original time periods
time_periods <- list(
 "Period1" = c(1, 50000),
 "Period2" = c(50000, 100000),
 "Period3" = c(100000, 200000),
 "Period4" = c(200000, 300000),
 "Period5" = c(300000, 400000),
 "Period6" = c(400000, 600000),
 "Period7" = c(600000, 2000000),
 "Period8" = c(2000000, 2800000),
 "Period9" = c(2800000, 4200000)
)

# Function to assign time periods
assign_time_period <- function(age, periods) {
 for (name in names(periods)) {
 bounds <- periods[[name]]
 if (!is.na(age) && age > bounds[1] && age <= bounds[2]) {
 return(name)
 }
 }
 return(NA)
}

length(levels(as.factor(dataset$First_Appearance_Age)))

[1] 51

# Assign original time periods
dataset$Original_Time_Period <- sapply(dataset$First_Appearance_Age, assign_time_period, periods = time_periods)

# Function to shift time periods by a factor
# Here we
shift_time_periods <- function(periods, shift_factor) {
 shifted_periods <- lapply(periods, function(bounds) c(bounds[1] * shift_factor[1], bounds[2] * shift_factor[2]))
 return(shifted_periods)
}

# Shift boundaries by +10%, -10%, +20%, and -20%
# Cannot change lower boundary by -10% and upper by +10% otherwise periods will overlap
time_periods_plus10 <- shift_time_periods(time_periods, c(1.1, 1.1))
time_periods_minus10 <- shift_time_periods(time_periods, c(0.9, 0.9))
time_periods_plus20 <- shift_time_periods(time_periods, c(1.2, 1.2))
time_periods_minus20 <- shift_time_periods(time_periods, c(0.8, 0.8))

# Assign shifted time periods
dataset$Shifted_Time_Period_plus10 <- sapply(dataset$First_Appearance_Age, assign_time_period, periods = time_periods_plus10)
dataset$Shifted_Time_Period_minus10 <- sapply(dataset$First_Appearance_Age, assign_time_period, periods = time_periods_minus10)
dataset$Shifted_Time_Period_plus20 <- sapply(dataset$First_Appearance_Age, assign_time_period, periods = time_periods_plus20)
dataset$Shifted_Time_Period_minus20 <- sapply(dataset$First_Appearance_Age, assign_time_period, periods = time_periods_minus20)

# Compute agreement percentages
agreement_plus10 <- mean(dataset$Original_Time_Period == dataset$Shifted_Time_Period_plus10, na.rm = TRUE) * 100
agreement_minus10 <- mean(dataset$Original_Time_Period == dataset$Shifted_Time_Period_minus10, na.rm = TRUE) * 100
agreement_plus20 <- mean(dataset$Original_Time_Period == dataset$Shifted_Time_Period_plus20, na.rm = TRUE) * 100
agreement_minus20 <- mean(dataset$Original_Time_Period == dataset$Shifted_Time_Period_minus20, na.rm = TRUE) * 100

# Print results
print(paste("Agreement with +10% shift:", round(agreement_plus10, 2), "%"))

[1] "Agreement with +10% shift: 92.23 %"

print(paste("Agreement with -10% shift:", round(agreement_minus10, 2), "%"))

[1] "Agreement with -10% shift: 80.58 %"

print(paste("Agreement with +20% shift:", round(agreement_plus20, 2), "%"))

[1] "Agreement with +20% shift: 79.61 %"

print(paste("Agreement with -20% shift:", round(agreement_minus20, 2), "%"))

[1] "Agreement with -20% shift: 61.17 %"

# Create contingency tables
contingency_table_plus10 <- table(dataset$Original_Time_Period, dataset$Shifted_Time_Period_plus10)
contingency_table_minus10 <- table(dataset$Original_Time_Period, dataset$Shifted_Time_Period_minus10)
contingency_table_plus20 <- table(dataset$Original_Time_Period, dataset$Shifted_Time_Period_plus20)
contingency_table_minus20 <- table(dataset$Original_Time_Period, dataset$Shifted_Time_Period_minus20)

# Perform Chi-square tests
chi2_plus10 <- chisq.test(contingency_table_plus10)
chi2_minus10 <- chisq.test(contingency_table_minus10)
chi2_plus20 <- chisq.test(contingency_table_plus20)
chi2_minus20 <- chisq.test(contingency_table_minus20)

# Print Chi-square test results
print("Chi-square test for +10% shift:")

[1] "Chi-square test for +10% shift:"

print(chi2_plus10)

Pearson's Chi-squared test

data: contingency_table_plus10
X-squared = 694.17, df = 64, p-value < 2.2e-16

print("Chi-square test for -10% shift:")

[1] "Chi-square test for -10% shift:"

print(chi2_minus10)

Pearson's Chi-squared test

data: contingency_table_minus10
X-squared = 570.81, df = 64, p-value < 2.2e-16

print("Chi-square test for +20% shift:")

[1] "Chi-square test for +20% shift:"

print(chi2_plus20)

Pearson's Chi-squared test

data: contingency_table_plus20
X-squared = 522.7, df = 56, p-value < 2.2e-16

print("Chi-square test for -20% shift:")

[1] "Chi-square test for -20% shift:"

print(chi2_minus20)

Pearson's Chi-squared test

data: contingency_table_minus20
X-squared = 448.9, df = 64, p-value < 2.2e-16

ggplot(dataset, aes(x = Original_Time_Period)) +
 geom_bar(fill = "blue", alpha = 0.7) + ylim(0,40) +labs(title = "Distribution of Cultural Traits' First Appearance (Original Periods)",
 x = "Original Time_Period of First Appearance", y = "Frequency")


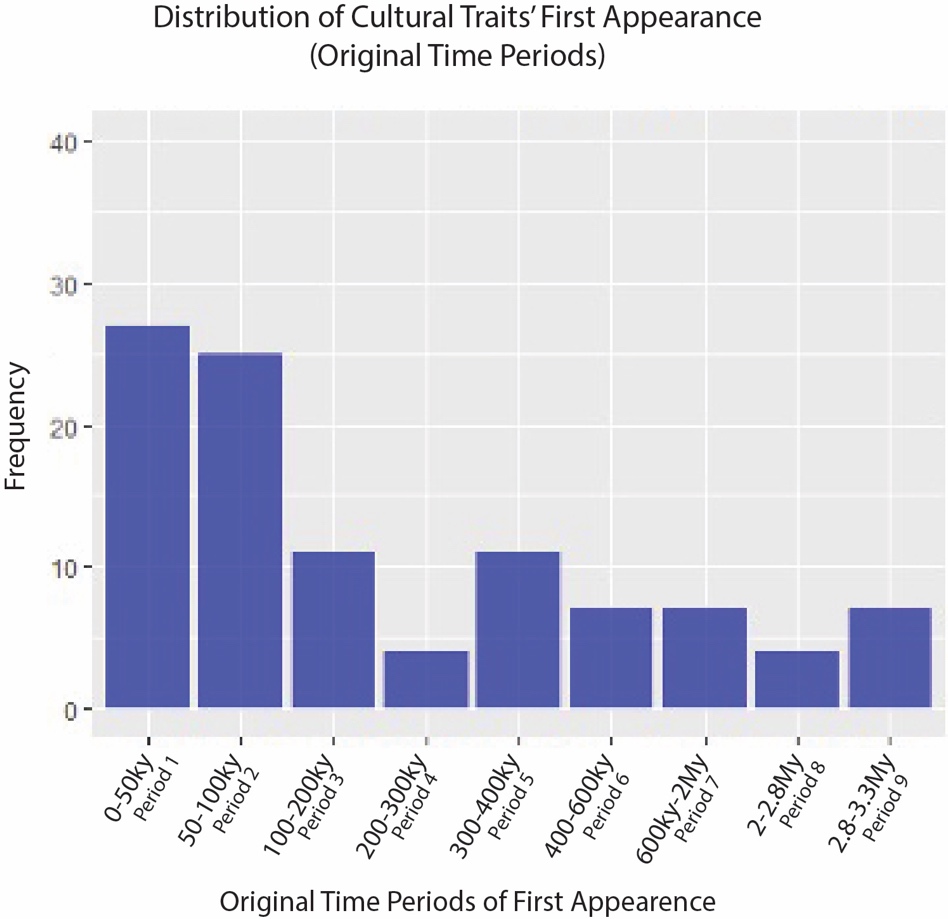


ggplot(dataset, aes(x = Shifted_Time_Period_plus10)) +
 geom_bar(fill = "blue", alpha = 0.7) + ylim(0,40) +labs(title = "Distribution of Cultural Traits' First Appearance (Periods +10%)",
 x = "Shifted_Time_Period_plus10 of First Appearance", y = "Frequency")


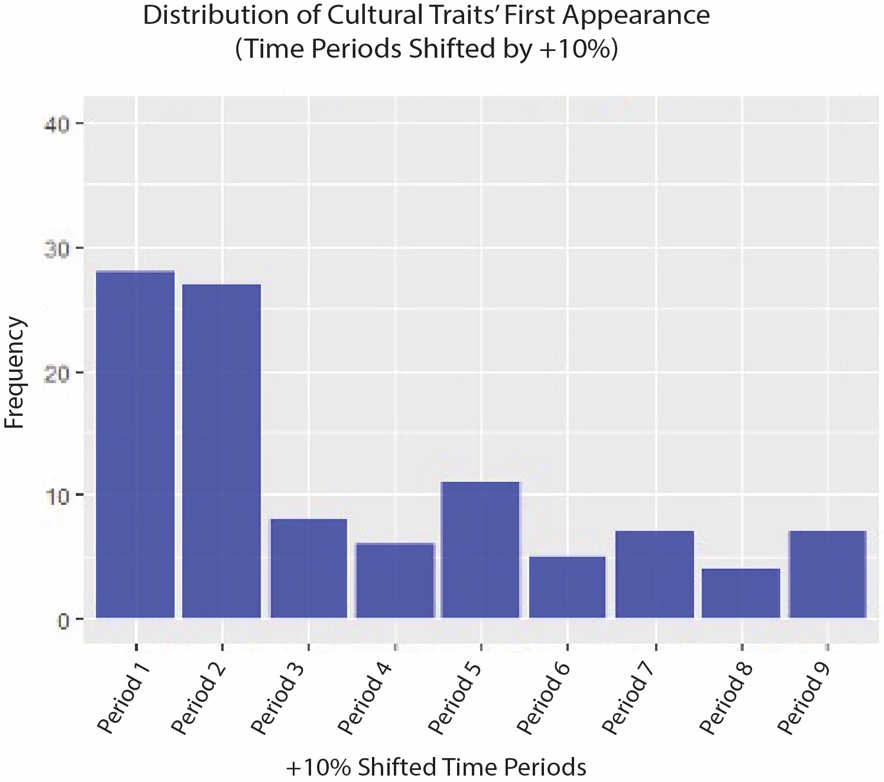


ggplot(dataset, aes(x = Shifted_Time_Period_minus10)) +
 geom_bar(fill = "blue", alpha = 0.7) + ylim(0,40) +labs(title = "Distribution of Cultural Traits' First Appearance (Periods -10%)",
 x = "Shifted_Time_Period_minus10 of First Appearance", y = "Frequency")


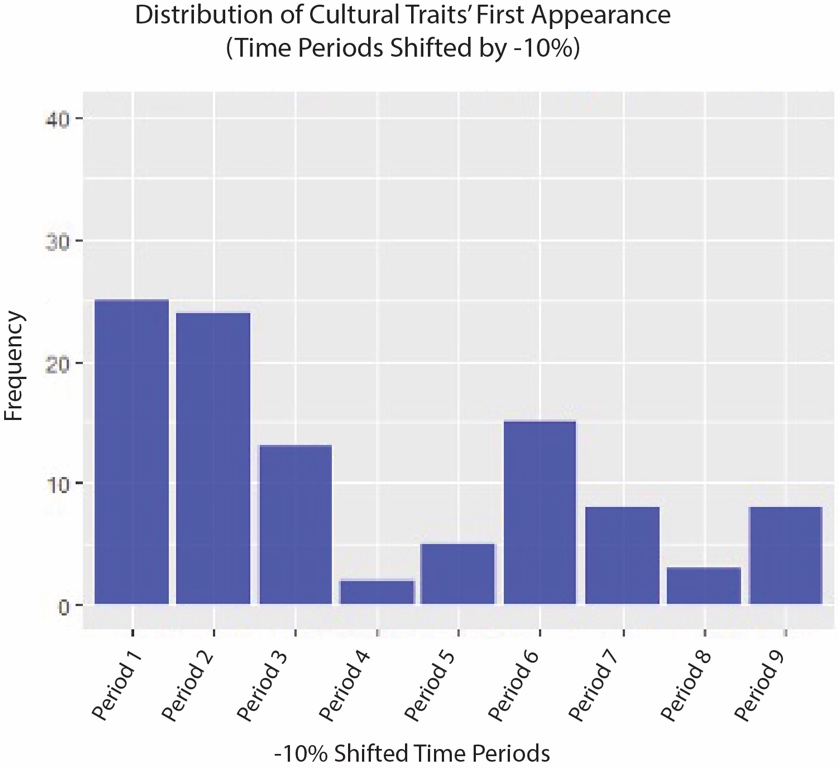


ggplot(dataset, aes(x = Shifted_Time_Period_plus20)) +
 geom_bar(fill = "blue", alpha = 0.7) + ylim(0,40) +labs(title = "Distribution of Cultural Traits' First Appearance (Periods +20%)",
 x = "Shifted_Time_Period_plus20 of First Appearance", y = "Frequency")


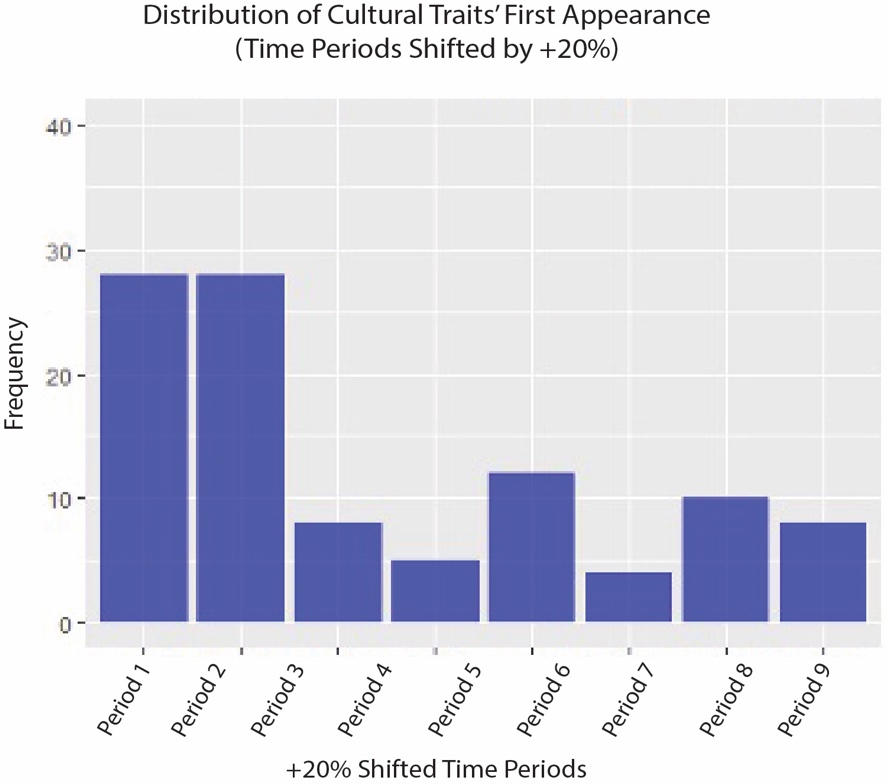


ggplot(dataset, aes(x = Shifted_Time_Period_minus20)) +
 geom_bar(fill = "blue", alpha = 0.7) + ylim(0,40) + labs(title = "Distribution of Cultural Traits' First Appearance (Periods -20%)",
 x = "Shifted_Time_Period_minus20 of First Appearance", y = "Frequency")


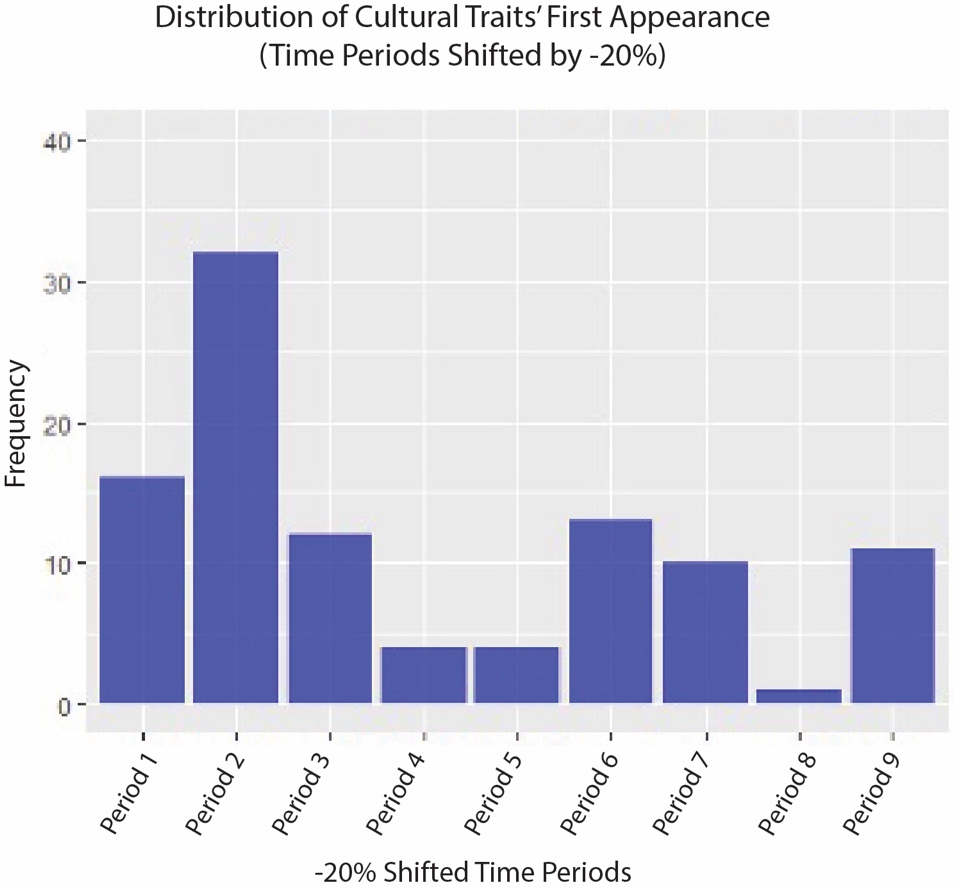


We can see that lowering the upper boundaries of periods has a stronger effect than lowering the lower limits. This is due to the fat that quite a number of cultural traits first appearance age are close to the upper limit of a period. Nevertheless, as per the chi-squared test, the original periods and any of the perturbed periods are not statistically independent.
